# Microenvironmental remodeling in FLASH radiotherapy

**DOI:** 10.3389/fcell.2026.1883109

**Published:** 2026-07-01

**Authors:** Yubo Zhao, Hang Li, Jiaqiao Zhong, Jianguo Xu, Liangxue Zhou, Yi Liu, Yuelong Wang

**Affiliations:** 1 Department of Neurosurgery, West China Hospital, Sichuan University, Chengdu, China; 2 State Key Laboratory of Biotherapy and Cancer Center, Research Unit of Gene and Immunotherapy, Chinese Academy of Medical Sciences, Collaborative Innovation Center of Biotherapy, West China Hospital, Sichuan University, Chengdu, Sichuan, China

**Keywords:** FLASH radiotherapy, immune microenvironment, microenvironmental remodeling, normal-tissue sparing, radiation biology, radiosensitization, redox biology, ultra-high dose rate radiotherapy

## Abstract

FLASH radiotherapy (FLASH-RT), characterized by ultra-high dose rate irradiation (>40 Gy/s), has emerged as a promising strategy for expanding the therapeutic window by sparing normal tissues while preserving tumor control. This review proposes a microenvironment-centered framework that interprets FLASH-RT as a physical-chemical-biological continuum: beam parameters and energy deposition shape oxygen dynamics and radical chemistry, which subsequently influence cellular stress responses, tissue repair, immune regulation, and late remodeling. Within this framework, we integrate current evidence regarding physical prerequisites, organ-specific normal-tissue responses, tumor-control outcomes, early clinical translation, and biological mechanisms. Preclinical studies across the central nervous system, lung, gastrointestinal tract, skin, and other organ systems suggest that FLASH-RT can attenuate acute inflammation, preserve stem and progenitor cell populations and regenerative capacity, reduce persistent DNA damage and cellular senescence, and mitigate late fibrosis. In several tumor models, FLASH-RT has maintained tumor control comparable to that achieved with conventional dose rate radiotherapy, without clear evidence of compromised antitumor efficacy. Early clinical studies have demonstrated feasibility and preliminary safety in superficial skin lesions and palliative treatment of bone metastases. Mechanistically, FLASH-induced microenvironmental remodeling may involve altered oxygen dynamics and radical chemistry, attenuation of inflammatory and fibrotic signaling, preservation of mitochondrial homeostasis, and modulation of immune responses. However, these responses are not uniform and depend on radiation modality, linear energy transfer, dose-rate structure, tissue context, and postirradiation time. By linking upstream irradiation conditions to downstream microenvironmental remodeling, this framework clarifies both shared responses and beam- or context-specific mechanisms. Standardized dosimetry, dynamic *in vivo* measurements, and paired tumor-normal tissue evaluations remain essential for testing causal relationships and guiding clinical translation.

## 1 Introduction

**FIGURE 1:**
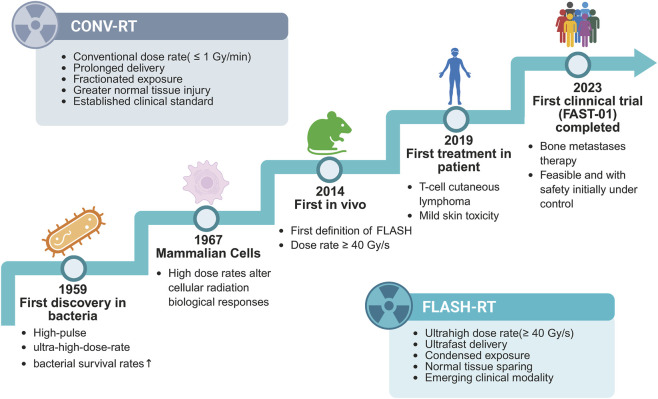
Key milestones in the development of FLASH radiotherapy and its distinction from conventional radiotherapy. The figure summarizes the major historical advances in FLASH radiotherapy, beginning with the early observation of ultra-high dose rate effects in bacteria (1959) and mammalian cells (1967), followed by the first *in vivo* demonstration and modern definition of FLASH as dose rates ≥40 Gy/s (2014), the first patient treatment (2019), and completion of the first clinical trial, FAST-01, in 2023. The figure also compares the key features of conventional radiotherapy (CONV-RT) and FLASH radiotherapy (FLASH-RT), highlighting differences in dose rate, delivery mode, fractionation pattern, treatment time, and normal tissue response.

Over the past decade, ultra-high dose rate radiotherapy (UHDR-RT), particularly FLASH radiotherapy, has evolved from a radiobiological observation into a translational strategy aimed at improving the therapeutic ratio of radiotherapy. Its principal appeal lies in the potential to maintain tumor control while selectively sparing normal tissues, thereby providing a rationale for reducing treatment-related toxicity, enabling dose escalation, and improving combination strategies (Favaudon et al., 2014). FLASH represents an emerging field at the intersection of radiation physics, chemistry, radiobiology, and clinical oncology. Its clinical implementation requires not only robust and verifiable dosimetry but also mechanistically interpretable biological readouts (Vozenin et al., 2024; Chow and Ruda, 2024; Rosini et al., 2025; Ma et al., 2024). As summarized in Figure 1, the development of FLASH radiotherapy has progressed from early high-dose-rate observations in bacteria and mammalian cells to *in vivo* validation, first-in-human application, and early clinical evaluation.

FLASH research is no longer confined to preclinical models. The proton FLASH (pFLASH) study in humans, FAST-01, demonstrated the feasibility and preliminary safety of a clinical treatment workflow (von der Grün et al., 2026; Lempart et al., 2019; Rahman et al., 2021). Notably, the central question in this field has shifted from whether UHDR irradiation can be technically delivered to the biological and clinical conditions under which such delivery can reproducibly generate therapeutic benefit. In parallel, electron FLASH (eFLASH) has been reported in an initial human application following modification of a conventional linear accelerator. Accordingly, the key questions now extend beyond feasibility to include the conditions required for consistent benefit, the extent to which microenvironmental features modulate these effects, and the manner in which such benefits should be validated using standardized dosimetric parameters and mechanistic endpoints (Chow and Ruda, 2023; Siddique et al., 2023).

Existing reviews have systematically discussed FLASH radiotherapy from the perspectives of clinical translation, mechanistic hypotheses, dosimetric challenges, and combination therapy. However, analyses focused solely on irradiation platforms, dosimetry, or early clinical progress remain insufficient to address several fundamental scientific questions in the field. To date, there is still a lack of an analytical review centered on microenvironmental alterations that integrates normal-tissue protection, tumor control, combination strategies, and translational requirements in FLASH radiotherapy. This gap is important because current evidence suggests that the differential effects of FLASH are unlikely to arise from a single isolated mechanism. Rather, they appear to reflect spatiotemporal remodeling across multiple biological levels, including local redox status, inflammatory amplification, modes of cell death, vascular and barrier integrity, regenerative cell ecology, metabolic homeostasis, and immune composition, all induced or modulated by UHDR irradiation. In this context, the translational value of FLASH may lie in its capacity to reshape the radiotherapeutic microenvironment by altering the responses of both normal tissues and the tumor microenvironment. It is therefore important to determine whether FLASH can expand the therapeutic window through microenvironmental remodeling and whether such changes are sufficient to support more rational treatment intensification and combination approaches.

This review summarizes current evidence regarding FLASH-induced microenvironmental changes within the broader context of FLASH development and clinical translation. First, we outline the physical prerequisites of the FLASH effect, with particular emphasis on its parametric and dosimetric characteristics. Second, we summarize the microenvironmental modules for which evidence is currently most concentrated, including redox status, inflammation and tissue barriers, regeneration and fibrosis, metabolism and mitochondrial homeostasis, and local and systemic immune ecosystems. Finally, we discuss potential radiosensitization and combination strategies shaped by these microenvironmental changes and further examine the translational requirements and evidentiary limitations of the current literature.

## 2 Physical prerequisites for FLASH-RT–induced microenvironmental changes

Electrons, protons, X-rays, and carbon ions have all been used in FLASH radiotherapy research. The fundamental differences among these radiation modalities lie not only in their delivery techniques but also in their linear energy transfer (LET), track structure, and the resulting initial radiochemical environment. Under FLASH conditions, early physicochemical processes—including microscopic patterns of energy deposition, radical generation and recombination, and oxygen-mediated reactions—are jointly determined by beam structure and temporal delivery characteristics. Therefore, FLASH-induced microenvironmental effects should first be understood as biologically relevant consequences of specific physical and dosimetric conditions, rather than as biological phenomena detached from their irradiation context. In particular, microdosimetric studies suggest that FLASH pulses may generate denser clusters of energy deposition at the submicrometer scale, thereby altering the spatial distribution of ionization events and potentially promoting local radical recombination. These processes provide a physical basis for subsequent microenvironmental differences (Ma et al., 2024; Tinganelli et al., 2025).

### 2.1 Radiation source–dependent differences in the radiochemical environment

In FLASH research, electrons, protons, X-rays, and carbon ions are often discussed collectively. However, their differences extend beyond penetration depth, equipment platforms, and clinical accessibility. These radiation modalities differ fundamentally in their patterns of initial energy deposition, thereby producing distinct radiochemical environments. The early physicochemical events currently implicated in FLASH—including water radiolysis, radical production and recombination, oxygen-mediated reactions, and subsequent oxidative chain reactions—are closely linked to radiation quality. Accordingly, FLASH effects observed with different radiation sources should not be compared in an overly simplified manner (Ma et al., 2024; Yang et al., 2025a).

From a radiochemical perspective, radiation quality primarily determines LET and track structure. At low LET, ionization events are spatially dispersed, and reactive species generated by water radiolysis are distributed relatively sparsely. Subsequent biological damage therefore depends largely on radical diffusion, oxygen interactions, and indirect damage mechanisms. As LET increases, ionization events become more densely clustered, local radical concentrations increase, the probability of radical recombination rises, and the contribution of direct damage becomes greater. Accordingly, oxygen-depletion and radical-recombination models, which were largely developed in low-LET settings, should not be treated as universal explanations. Experimental and track-structure studies indicate that oxygen consumption depends on dose, dose rate, and LET, but clinically relevant doses do not fully deplete oxygen. Transient oxygen depletion is therefore unlikely to represent a single unifying mechanism of the FLASH effect (Ali and El Naqa, 2025; Boscolo et al., 2021; Griffin, 2006).

As summarized in Table 1, electrons and X-rays are both low-LET modalities, but their energy-deposition patterns are not identical: X-ray effects are mediated through secondary electrons, whereas primary electron beams provide a comparatively direct low-LET model. Proton beams introduce depth-dependent heterogeneity because LET increases toward the distal Bragg-peak region. Carbon ions provide a high-LET contrast, with denser tracks, more clustered ionization events, and a larger contribution of direct damage. Direct electron-proton comparisons also show similar yields of short-lived ROS but differences in longer-lived reactive species, indicating that nominally low-LET modalities are not necessarily radiochemically interchangeable (Kalholm et al., 2021; Thomas et al., 2024; Suit et al., 2010; Tinganelli et al., 2022a; Tinganelli et al., 2022b).

**TABLE 1:** Radiation modality-dependent radiochemical features and implications for microenvironmental remodeling in FLASH radiotherapy.

Modality	LET/track structure	Radiochemical feature	Potential microenvironmental implication	Advantage
Electrons	Low LET; relatively sparse tracks	Useful low-LET model for studying radical diffusion, recombination, and oxygen-dependent chemistry	May reduce oxidative injury and downstream inflammatory or fibrotic amplification under appropriate delivery conditions	Accessible UHDR platforms and the largest preclinical evidence base. Findings should not be extrapolated unchanged to higher-LET beams
X-rays/photons	Low LET; energy is deposited mainly through secondary electrons	Radiochemistry depends on photon spectrum, depth, and secondary-electron distribution	May alter oxidative stress, inflammation, and barrier injury, but pathway-level equivalence with electrons requires validation	Greater penetration and clinical familiarity. UHDR generation and dosimetry remain technically challenging
Protons	Low-to-intermediate LET; LET increases toward the distal Bragg-peak region	Depth-dependent LET creates spatially heterogeneous radical chemistry	Redox signaling, inflammation, and repair may vary across depth and cannot be inferred from a field-average dose rate alone	Favorable depth-dose profile and pencil-beam scanning. Transmission-beam findings should not be directly extrapolated to Bragg-peak conditions
Carbon ions	Higher LET; dense and clustered tracks. LET varies with depth	High local ionization density increases direct damage and short-range radical interactions	May produce distinct redox, cell-death, inflammatory, and immune responses; tissue sparing can depend on LET and dose rate	High conformality and biological effectiveness. Current FLASH evidence remains limited; low-LET models cannot be directly applied

Accordingly, when evaluating the effects of FLASH on redox regulation, inflammation, tissue barriers, regeneration, or immune responses, electrons, protons, X-rays, and carbon ions should not be treated as interchangeable FLASH conditions. Similar biological phenotypes may arise from nonidentical upstream processes. Mechanistic interpretation and combination strategies should therefore be grounded in explicitly reported beam characteristics, LET or LET distributions, dose-rate structure, and temporal parameters. This approach improves dosimetric auditability and prevents unsupported cross-modality extrapolation.

### 2.2 Multivariable control as a prerequisite for FLASH-RT–induced microenvironmental effects

Current evidence does not support the view that the FLASH effect is a single phenomenon governed by a uniform average dose-rate threshold. Instead, FLASH is increasingly understood as a context-dependent biological response shaped by multiple interrelated parameters, including average dose rate, dose per fraction, total irradiation time, pulse structure, spatial dose-rate distribution, beam type, and tissue background (Vozenin et al., 2024; Vozenin et al., 2022). Although dose rate remains a central parameter in FLASH radiotherapy, it represents only one component of a multidimensional delivery framework. When interpreted in isolation from total delivery time and temporal beam structure, its explanatory value is limited. Recent UHDR/FLASH reporting recommendations have therefore emphasized intra-pulse dose rate, pulse width, pulse interval, and three-dimensional dose-rate coverage as essential variables for documentation and auditing (Tobias Böhlen et al., 2024; Schneider et al., 2025). These requirements underscore that FLASH should not be defined solely by achieving a specific average dose-rate value. Pulse shape and instantaneous dose rate can influence ionization density and track structure, thereby affecting radical formation, radical recombination, and oxygen consumption kinetics—processes that are closely linked to the FLASH effect (Esplen et al., 2020; Yang et al., 2023).

Current *in vivo* studies further indicate that dose per fraction and fractionation schedule substantially influence the detectability and magnitude of the FLASH effect. Normal-tissue sparing is more consistently observed at high single doses and with hypofractionated delivery. In contrast, as dose per fraction decreases and the number of fractions increases, the protective effect generally diminishes, although the extent of this decline varies across organs. Systematic reviews and modeling studies suggest that tissues such as skin and intestine may be particularly sensitive to increasing fraction number, whereas brain tissue may retain some degree of functional protection under selected hypofractionated regimens (Montay-Gruel et al., 2017; Ruan et al., 2021; Montay-Gruel et al., 2021). These findings suggest that FLASH is more likely to confer a normal-tissue advantage under specific conditions involving high dose per fraction and limited fraction numbers (Sesink et al., 2026; Kristensen et al., 2025a). Thus, dose rate, dose per fraction, and fractionation should not be considered independently; rather, the FLASH effect appears to depend on their interaction (Grilj et al., 2025).

The importance of the parameter window lies in its role as the physical foundation of FLASH-induced microenvironmental effects. It should not be viewed as a purely technical condition separate from mechanism (Rohrer Bley et al., 2022). Current discussions of FLASH—including redox modulation, inflammatory regulation, barrier protection, preservation of regenerative cell populations, and immune microenvironment remodeling—implicitly assume that the initial radiation-induced chemical and injury signals occur within defined temporal and spatial boundaries. Radiation-induced oxygen depletion alone cannot fully explain all reported FLASH phenomena. A more plausible interpretation is that the initial radiochemical environment becomes biologically consequential only when average dose rate, dose per fraction, temporal pulse structure, and spatial dose-rate coverage fall within an appropriate range. Therefore, mechanistic studies of FLASH-induced microenvironmental remodeling must be based on well-defined parameter windows and verifiable dosimetry. Without such standardization, biological differences observed across studies will remain difficult to compare, interpret, and translate.

## 3 Tissue hierarchy remodeling in FLASH-RT animal models and preservation of tumor control

### 3.1 Changes in the normal tissue microenvironment

**FIGURE 2:**
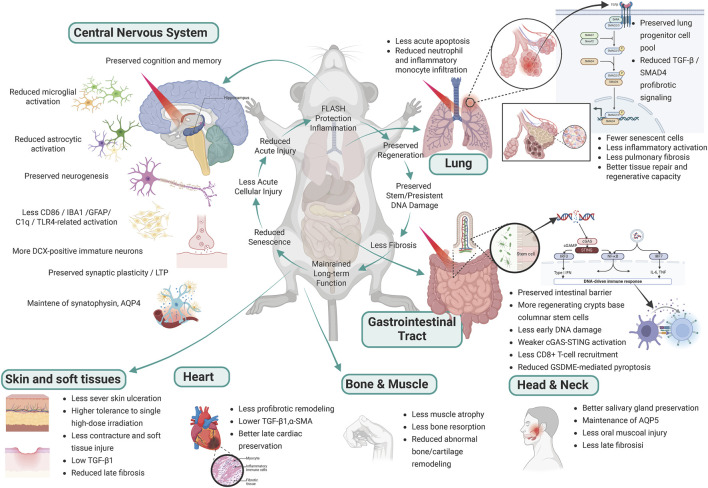
Organ-specific normal tissue protection associated with FLASH radiotherapy. This schematic summarizes the current preclinical evidence that FLASH radiotherapy confers normal tissue protection across multiple organ systems. The central protective theme includes reduced acute cellular injury, attenuation of inflammatory activation, preservation of regeneration and progenitor/stem cell pools, reduced persistent DNA damage and senescence, less fibrosis, and maintenance of long-term tissue function. In the central nervous system, FLASH is associated with preservation of cognition and memory, reduced microglial and astrocytic activation, preserved neurogenesis, and maintenance of synaptic plasticity. In the lung, FLASH is linked to reduced acute apoptosis and inflammatory cell infiltration, preservation of the lung progenitor cell pool, attenuation of TGF-β/SMAD4-related profibrotic signaling, fewer senescent cells, and reduced pulmonary fibrosis. In the gastrointestinal tract, FLASH preserves intestinal barrier integrity and crypt regeneration, reduces early DNA damage, weakens cGAS–STING activation and CD8^+^ T-cell recruitment, and suppresses GSDME-mediated pyroptosis. Additional protective effects have also been reported in skin and soft tissues, the heart, bone and muscle, and head and neck organs, although the strength and consistency of evidence vary by organ system and endpoint.

Figure 2 summarizes the normal-tissue effects of FLASH radiotherapy across multiple organ systems. These effects converge on several recurring biological themes, including reduced acute cellular injury, attenuated inflammatory responses, preservation of stem and progenitor cell populations and regenerative capacity, decreased persistent DNA damage and cellular senescence, reduced fibrosis, and improved long-term functional maintenance. The strength of evidence varies across organ systems, with the most robust preclinical support currently reported in the central nervous system (CNS), lung, and gastrointestinal tract.

#### 3.1.1 Central nervous system

Preclinical studies in the central nervous system support the hypothesis that FLASH radiotherapy can protect normal brain tissue. This protection is reflected by preserved neurocognitive function, reduced neuroinflammation, attenuated impairment of neurogenesis, maintenance of synaptic plasticity, and decreased radiation-induced injury to the neurovascular and perivascular microenvironment. Recent preclinical studies of FLASH in brain irradiation models, including both normal-brain protection and brain tumor settings, are summarized in Table 2.

**TABLE 2:** Representative preclinical studies of FLASH radiotherapy in brain irradiation models.

Year	Radioactive particles	Radiation method	Dose and dose rate	Main outcome measures	Model	References
2024	Electron	Whole-Brain Irradiation (WBI)	10 Gy (5.6 × 10^6 Gy/s)	Neuronal Synaptic Myelin	Normal Mouse	Dickstein et al. (2024)
2024	Electron	Two Lateral Beams Hemibrain Irradiation (HBI)	40 MeV (115 Gy/s)	Dose rate Dose uniformity Electron beam parameters	Monte Carlo Simulations Pediatric WBI	Brei and tkreutz (2020)
2024	Helium	HBI	10 Gy (141 Gy/s)	DNA damage Brain microvascular integrity Microglia/Macrophage activation	Normal Mouse	Dokic et al. (2024)
2023	Electron	WBI	3 × 10 Gy (5.6 × 10^6 Gy/s)	Cognitive function Synaptic Neuroinflammation	Normal Mouse	Alaghband et al. (2023)
2023	Electron	WBI	3 × 10 Gy (1.6 × 10^6 Gy/s)	Synaptic plasticity Synaptic transmission	Normal Mouse	Limoli et al. (2023)
2023	Proton	HBI	25 Gy (257 ± 2 Gy/s)	Cognitive function Peripheral immune cells Neuroinflammatory Immune cell infiltration	Glioma Mouse Model	Iturri et al. (2023)
2023	Electron	HBI	20 Gy; 25 Gy; 30 Gy (≥429 Gy/s)	Survival rate Tumor size Cutaneous side effects	Glioblastoma Mouse Model	Liljedahl et al. (2023)
2023	Electron	WBI	2 × 10 Gy (5.6 × 106 Gy/s)	Synaptic Cognitive function Neuroinflammation Cerebrovascular integrity	Juvenile Mice	Allen et al. (2023)
2022	Electron	HBI	Subcutaneous: 2 × 8 GyIntracranial: 2 × 12.5 Gy (66 Gy/s)	Tumor long-term resistance Survival rate Tumor size Serum analytes Gene expression	Glioblastoma Mouse Model	Liljedahl et al. (2022)
2022	Proton	9 Spots HBI	10 Gy (120 Gy/s)	DNA damage Brain microenvironment impairment Inflammatory microenvironment	Normal Mouse	Dokic et al. (2022)
2022	Electron	HBI	3 × 8 Gy; 3 × 12.5 Gy; 3 × 15 Gy (70–90 Gy/s)	Long-term tumor control rate Survival rate Cutaneous toxicity	Glioma Mouse Model	Konradsson et al. (2022)
2021	Electron	WBI&HBI	WBI: 10 Gy; 14 Gy; 4 × 3.5 Gy; 2 × 7 Gy; 3 × 10 Gy HBI:25Gy (1.8 × 106 Gy/s)	Tumor growth Cognitive function Survival rate	Glioblastoma Mouse Model	Montay-Gruel et al. (2021)
2020	Electron	WBI	25 Gy; 10 Gy (2; 500 Gy/s)	Vascular volume eNOS expression Tight junction proteins Cell apoptosis	Normal Mouse	Allen et al. (2020)
2020	Electron	WBI	10 Gy (106 Gy/s)	Neuroinflammation Complement system activation	Normal Mouse	Montay-Gruel et al. (2020)
2020	Electron	WBI	8Gy (4.4 × 10^6 Gy/s)	Cognitive function Neurogenesis Neuroinflammation Endocrine regulation	Juvenile Mouse	Alaghband et al. (2020)
2019	Electron	WBI	10Gy (>1.8 × 10^6 Gy/s)	Cognitive function Neuroinflammation Neuronal structure	Normal Mouse	Montay-Gruel et al. (2019)
2018	X-ray	WBI	10 Gy (37 Gy/s)	Cognitive function Hippocampal cell Neuroinflammation	Normal Mouse	Montay-Gruel et al. (2018)
2017	Electron	WBI	10 Gy (1; 3; 10; 30; 60; 100; 500 Gy/s; 5.6 MGy/s)	Cognitive function Neurogenesis	Normal Mouse	Montay-Gruel et al. (2017)

In adult mice, Montay-Gruel et al. delivered a single 10 Gy dose of whole-brain irradiation and showed that FLASH irradiation (>100 Gy/s) did not induce the learning and memory deficits observed after conventional dose rate irradiation. In contrast, mice in the conventional irradiation group exhibited persistent neurocognitive impairment at 6 months (Montay-Gruel et al., 2019). Similar findings were reported after 30 Gy whole-brain irradiation. At 10 weeks after treatment, the conventional irradiation group showed impaired behavioral and cognitive performance, whereas the FLASH group performed similarly to nonirradiated controls. Moreover, conventional irradiation was associated with reduced hippocampal dendritic spine density, increased CD68^+^ activated microglia, and greater upregulation of inflammatory markers (Simmons et al., 2019). These findings indicate that the effects of FLASH on the brain extend beyond behavioral preservation and involve alterations in synaptic architecture and the neuroinflammatory microenvironment. At present, these tissue-level observations should be interpreted as reproducible features of low-LET electron FLASH models rather than as established cross-modality endpoints.

This differential effect has also been observed in juvenile animals. Alaghband et al. administered a single 8 Gy dose of whole-brain irradiation to 3-week-old mice. The conventional dose rate group (0.077 Gy/s) exhibited persistent deficits in spatial memory and novel object recognition at 2–4 months, whereas the FLASH group (4.4 × 10^6^ Gy/s) showed significant preservation of these functions. Novel object recognition analysis demonstrated a significant overall difference among groups, with poorer performance in the conventional irradiation group than in both the control group and the FLASH group. Histologically, conventional irradiation reduced the number of DCX^+^ immature neurons and the proportion of BrdU^+^/NeuN^+^ neurogenic cells, while increasing microglial activation, as indicated by CD68/IBA1 staining. These alterations were substantially attenuated in the FLASH group (Alaghband et al., 2020). Thus, the benefit of FLASH in the developing brain appears to extend beyond functional preservation and is associated with reduced disruption of neurogenesis, synaptic integrity, and glial/perivascular units.

FLASH-induced changes in brain tissue are also evident at the levels of glial homeostasis, synaptic function, and neurovascular integrity. After a single 10 Gy dose of whole-brain irradiation, conventional dose rate irradiation induced marked hypertrophy of GFAP^+^ astrocytes and stronger C1q/TLR4-related responses, whereas the FLASH group remained closer to the nonirradiated state. In fractionated whole-brain irradiation models using 2 × 10 Gy or 3 × 10 Gy regimens, FLASH continued to reduce long-term cognitive impairment and preserved long-term potentiation, synaptophysin expression, AQP4 distribution, and perivascular microenvironmental homeostasis (Allen et al., 2023; Alaghband et al., 2023). A 2024 multicenter study further validated the protective effects of single-dose 10 Gy electron FLASH whole-brain irradiation, demonstrating preservation of novel object recognition at 4 months and long-term potentiation at 5 months across multiple institutions and irradiation platforms. However, these protective effects were not consistently observed at the earliest time points, suggesting that the advantages of CNS-directed FLASH may be most evident in delayed recovery and long-term functional maintenance and may be accompanied by tissue-level differences in neuroinflammation and neurogenesis (Drayson et al., 2024).

Taken together, current evidence suggests that FLASH can mitigate delayed brain injury in mouse models of whole-brain irradiation. This conclusion is best supported for low-LET electron irradiation, with additional proof-of-concept support from low-LET synchrotron X-rays; it should not yet be presented as a modality-independent property of proton or high-LET carbon-ion beams. The 2024 multicenter study replicated these findings in a 10 Gy whole-brain electron FLASH model, showing protection of novel object recognition at 4 months and long-term potentiation at 5 months, thereby strengthening the reproducibility of this conclusion (Montay-Gruel et al., 2019).

Nevertheless, the current evidence more strongly supports the view that FLASH alters the trajectory of microenvironmental evolution in irradiated brain tissue than the conclusion that its protective mechanism has been definitively established. For example, it remains unclear whether this protection is driven primarily by oxygen depletion, altered radical chemistry, or remodeling of immune and glial cell responses. Oxygen-dependent chemistry, radical recombination, and subsequent inflammatory remodeling are plausible partially shared processes across low-LET FLASH modalities, but their relative contributions are expected to vary with radiation quality, LET, pulse structure, dose per pulse, and tissue oxygenation. They should therefore be described as candidate common mechanisms rather than universally established pathways. Furthermore, early molecular changes do not always correlate with late functional preservation, and inflammatory mediators such as complement C1q/C3 are not completely suppressed by FLASH in some studies. These findings suggest that the role of FLASH should not be simplistically characterized as purely anti-inflammatory (Montay-Gruel et al., 2020).

Several limitations should also be acknowledged. Existing studies have primarily used mouse models of whole-brain irradiation, high single doses, or a limited range of hypofractionated regimens. Across studies, there is considerable heterogeneity in animal age, sex, irradiated brain region, anesthesia method, and timing of behavioral assessment. In addition, FLASH delivery itself is influenced by average dose rate, instantaneous dose rate, pulse structure, total irradiation time, radiation modality, and methods of dosimetric verification. Therefore, current conclusions remain conditional and context dependent. Future studies should further define dose-rate thresholds, evaluate the feasibility and biological consequences of fractionated FLASH irradiation, clarify differences across brain regions and developmental stages, confirm whether tumor control is preserved, and identify microenvironmental biomarkers that can reliably predict long-term neuroprotective effects. Cross-modality studies should report beam type, energy, LET or LET distribution where applicable, dose per pulse, pulse structure, and irradiation time, and should test whether candidate downstream pathways are shared across low-LET and high-LET irradiation or are restricted to specific beam conditions.

#### 3.1.2 Lung

At the tissue level, FLASH-induced changes in the lung are primarily characterized by reduced acute cellular injury and inflammation, preservation of regenerative cell populations, decreased persistent DNA damage and cellular senescence, and attenuation of late fibrosis and structural remodeling. Favaudon et al. conducted a foundational study in C57BL/6J mice using single-dose bilateral thoracic irradiation (low-LET electron FLASH), involving a total of 240 animals. Their findings showed that conventional dose rate irradiation (CONV) at 15 Gy was sufficient to induce pulmonary fibrosis, whereas 17 Gy delivered with FLASH produced no histological evidence of pulmonary fibrosis or activation of the TGF-β/SMAD4 pathway. Subsequent dose escalation further demonstrated that pulmonary fibrosis was not observed within 36 weeks after 20 Gy FLASH, and marked pulmonary edema and fibrotic foci appeared only 24 weeks after 30 Gy FLASH. With respect to acute injury, 7.5 Gy CONV induced substantial caspase-3 cleavage at 1 h after irradiation and TUNEL positivity at 24 h, whereas 17 Gy FLASH produced minimal apoptotic signals. A dose of 30 Gy FLASH was required to induce apoptosis at a level comparable to that observed after 7.5 Gy CONV. *In vivo* annexin V imaging further showed that, in the absence of TNF-α stimulation, the apoptotic fluorescence signal after 30 Gy FLASH remained approximately twofold lower than that after 15 Gy CONV (Favaudon et al., 2014). These findings suggest that the protective effect of low-LET electron FLASH is not restricted to late fibrotic endpoints but is already evident during the early injury phase.

Subsequent studies examined this phenomenon in greater mechanistic detail and linked it to preservation of lung progenitor cells, reduced persistent DNA damage, and attenuation of cellular senescence. In a bilateral thoracic low-LET 4.5-MeV electron irradiation model, integrated qPCR, single-cell RNA sequencing, and histological analyses showed that FLASH, compared with CONV, reduced pro-inflammatory gene activation and mitigated the aberrant proliferative response of lung progenitor cells after injury. At later time points, FLASH-treated animals exhibited fewer persistent DNA damage foci and fewer senescent cells. These protective effects were lost in Terc^−/−^ mice with impaired telomerase activity, suggesting that the lung-sparing effect of electron FLASH depends, at least in part, on preservation of the replicative capacity of regenerative cell populations (Fouillade et al., 2020). Therefore, one of the most important tissue-level effects of FLASH in the lung may not be merely suppression of inflammation, but rather preservation of the progenitor-cell compartment and regenerative microenvironment required for repair, thereby limiting the progression toward cellular senescence and fibrosis.

Recent studies have further supported the temporal and hierarchical continuity of this response. A 2025 single-cell study using 17.8 Gy whole-thorax irradiation with a 6-MeV electron beam showed that, during the acute phase within 1 week after irradiation, FLASH induced a more rapid but transient pulmonary response, whereas CONV produced sustained inflammation. Histological scoring, immunostaining, and molecular assays indicated that FLASH significantly reduced neutrophil infiltration, particularly the pro-inflammatory Ccrl2^+^ subset, while attenuating the pro-inflammatory activation of Mefv^+^ monocytes and enhancing repair-associated TGF-β/epithelial–mesenchymal transition signaling in alveolar epithelial type 1 cells (Lu et al., 2025). It is important to note that this acute epithelial response should be distinguished from persistent TGF-β/SMAD activation in fibroblasts and myofibroblasts during the late remodeling phase, which is associated with extracellular-matrix deposition and fibrosis. In addition, a study using patient-derived lung tissue slices showed that, among 19 human lung samples, FLASH preserved a greater proportion of proliferating cells in 18 samples. RNA sequencing further confirmed reduced activation of pathways associated with cell-cycle arrest, p53-mediated apoptosis, DNA damage, and oxidative stress responses (Dubail et al., 2025).

However, this sequence is currently supported mainly by low-LET electron studies. It may represent a common framework for lung sparing, but its quantitative thresholds and downstream signatures cannot be directly extrapolated to proton beams or high-LET carbon ions, because LET-dependent track structure can alter initial ionization clustering and radical chemistry. The specific microenvironmental determinants responsible for lung protection remain incompletely defined. In particular, the potential stage-dependent dual roles of TGF-β/epithelial–mesenchymal transition signaling in tissue repair and fibrotic remodeling require further clarification. Recent single-cell and human lung tissue slice studies have strengthened the translational relevance of these findings. Nevertheless, differences in sample size, model systems, dose rate and pulse structure, radiation modality, dosimetric verification, and observation period continue to limit the generalizability of current conclusions. Future studies should further define dose-rate thresholds, evaluate the efficacy and feasibility of fractionated FLASH irradiation, confirm whether tumor control is preserved, and identify early biomarkers that can reliably predict late lung protection.

#### 3.1.3 Gastrointestinal and abdominal organs

In the gastrointestinal tract and abdominal organs, FLASH radiotherapy induces a series of interrelated changes in the tissue microenvironment. These changes are characterized by preservation of crypt stem-cell niches and regenerative capacity, reduced disruption of the mucosal barrier, attenuation of local inflammatory amplification, and decreased programmed cell death in specific inflammatory cell populations. The most reproducible evidence for these endpoints has been obtained with low-LET electron beams. Levy et al. first showed that whole-abdomen irradiation with a low-LET 16-MeV electron beam at a dose of 16 Gy delivered at a conventional dose rate resulted in 100% mortality in mice. In contrast, most mice receiving the same dose under FLASH conditions survived. At 96 h after irradiation, the FLASH group exhibited more than twice the number of regenerating crypts observed in the conventional dose rate group. At a sublethal dose of 14 Gy, intestinal functional recovery was also accelerated in the FLASH group. The conventional dose rate group showed a marked increase in serum FITC-dextran levels at 96 h, indicating increased intestinal barrier permeability, whereas the FLASH group remained close to the nonirradiated control. Histologically, the number of regenerating crypts increased 2.4-fold in the FLASH group compared with the conventional dose rate group after 14 Gy irradiation, with a similar trend observed after 12 Gy. Further mechanistic analyses showed that electron FLASH reduced early DNA damage and cell death in crypt basal columnar cells (Levy et al., 2020). These findings suggest that the gastrointestinal effects of low-LET electron FLASH are primarily reflected in preservation of intestinal epithelial stem-cell niches and maintenance of regenerative capacity.

This protective effect is time dependent, reproducible across institutions, and indicative of a distinct trajectory of microenvironmental remodeling after injury. Using a mouse model of crypt survival after whole-abdomen irradiation, Ruan et al. used a 6-MeV electron linear accelerator and found that, within a dose range of 7.5–12.5 Gy, the dose-modification factor of FLASH for acute intestinal toxicity was approximately 1.1. At 11.2 Gy, the proportion of surviving crypts after single-pulse FLASH was 27.6% ± 4.0%. However, when delivery was divided into two pulses and the average dose rate was reduced to 280 Gy/s, the proportion of surviving niches decreased to 6.9% ± 2.8%. These findings suggest that the gastrointestinal FLASH effect diminishes as total delivery time increases and average dose rate decreases (Ruan et al., 2021). An average dose rate of at least 280 Gy/s appears to be required to achieve robust intestinal protection. A multicenter comparative study by Zayas et al. further showed that, when key beam parameters were harmonized, the gastrointestinal FLASH effect was highly reproducible. At 15.5 Gy and 17 Gy, 30-day survival rates were higher in the FLASH group than in the conventional dose rate group. The FLASH group also exhibited prolonged survival, and the number of surviving crypts at 48–72 h after irradiation was significantly greater than that in the conventional dose rate group (Valdés Za et al., 2023).

Recent mechanistic studies have further extended this protective effect to inflammatory microenvironment remodeling. Shi et al. showed that FLASH significantly reduced radiation-induced colitis in mice receiving 13 Gy whole-abdominal X-ray irradiation combined with PD-L1 blockade. This protective effect was associated with reduced cytoplasmic double-stranded DNA accumulation in intestinal crypts, attenuated cGAS–STING activation, decreased CD8^+^ T-cell chemotaxis, and reduced GSDME-mediated pyroptosis (Shi et al., 2022). This study expands the mechanistic understanding of FLASH-mediated gastrointestinal protection beyond crypt regeneration alone and suggests that FLASH may also suppress immune- and inflammation-driven secondary tissue injury. However, the available evidence is still concentrated primarily in small-intestine and whole-abdomen irradiation models, whereas direct protective evidence for other abdominal parenchymal organs, such as the liver, kidneys, and pancreas, remains limited (Levy et al., 2020; Shi et al., 2022).

Nevertheless, current conclusions remain provisional. It has not yet been fully determined whether gastrointestinal protection is driven primarily by reduced early DNA damage, preservation of stem-cell niches, immune-inflammatory remodeling, or a combination of these mechanisms. Cross-modality findings also remain heterogeneous: partial-abdominal and whole-abdominal proton FLASH studies have not consistently reproduced intestinal sparing and have in some settings reported no benefit or increased acute toxicity, whereas a recent 290-MeV/u carbon-ion study found protection only under high-LET conditions and dose rates exceeding 100 Gy/s (Zh et al., 2023; Cao et al., 2025). These results reinforce that electron-derived mechanistic conclusions cannot be transferred directly to proton or carbon-ion beams. Future studies should clarify the effects of fractionated FLASH irradiation, radiation modality, tumor control, microbiome-related modulation, and early biomarkers capable of predicting long-term gastrointestinal protection.

#### 3.1.4 Skin and soft tissue

At the tissue level, the primary effects of FLASH radiotherapy in skin and soft tissue include reduced acute superficial injury, attenuated local inflammatory and fibrosis-related responses, and improvement in selected late soft-tissue remodeling endpoints. The established evidence is derived mainly from low-LET electron and proton beams; recent carbon-ion data support acute skin sparing under a defined LET condition but do not establish identical downstream mechanisms across radiation modalities. Previous studies have shown that, in a low-LET 16-MeV electron hemithoracic irradiation model, within a single-fraction dose range of 30–40 Gy, conventional dose rate irradiation was associated with a higher incidence of severe skin ulceration and toxicity-related early termination, whereas severe toxicity was substantially reduced after FLASH irradiation. For example, at the highest dose level, ulceration was observed in all animals in the conventional dose rate group, and most reached the maximum toxicity score. In contrast, ulceration in the FLASH group was generally limited to approximately 40% of animals. Survival outcomes were also improved: within the 30–40 Gy dose range, median survival in the FLASH group remained greater than 180 days, compared with approximately 52–100 days in the conventional dose rate group (Shi et al., 2022). These findings suggest that FLASH can increase the tolerance of skin to high-dose single-fraction irradiation.

These benefits are not confined to the acute phase; a consistent trend has also been observed in late soft-tissue responses. However, the evidence should be separated by beam type. In a 250-MeV proton pencil-beam-scanning model using the plateau region, a single 35 Gy exposure reduced skin and plasma TGF-β1 levels and attenuated leg contracture and soft-tissue injury during follow-up. A separate dose-response study using 16-MeV electrons reported dose-modification factors of approximately 1.45–1.54 for acute skin injury and approximately 1.15 for late fibrosis (Cunningham et al., 2021; Kristensen et al., 2025b). These findings indicate that the protective effect of FLASH in skin and soft tissue is most pronounced during the acute phase, while a more modest benefit may persist in late fibrotic remodeling.

However, skin and soft-tissue models also illustrate important limitations of the FLASH effect. Large-animal and translational studies indicate that the beneficial effects of FLASH on skin and soft tissue are strongly influenced by irradiation volume and delivery technique. When delivered to smaller volumes as a single continuous exposure, FLASH reduces superficial toxicity, providing an important translational basis for normal-skin sparing (Vozenin et al., 2019a; Wilson et al., 2020; Vozenin et al., 2019b). However, when the irradiation volume is increased, acute toxicity may remain relatively mild, whereas severe late skin necrosis can develop and persist for 7–9 months. In randomized studies involving cats and minipigs, late maxillary necrosis occurred in three of seven animals in the FLASH group (43%) but in 0 of nine animals in the conventional fractionated irradiation group. These findings suggest that optimal normal-skin and soft-tissue sparing with FLASH is most likely to occur under conditions of limited irradiation volume, single-beam delivery, and short treatment duration. Nevertheless, these benefits are markedly attenuated under conditions involving large treatment volumes or complex delivery geometries (Rohrer Bley et al., 2022).

These endpoints have been reproduced in low-LET electron and proton models. A recent carbon-ion pencil-beam-scanning study further reported reduced acute skin reactions in mouse hindlimbs at the beam entrance region (dose-averaged LET approximately 13 keV/μm; FLASH average dose rate approximately 167–191 Gy/s), with an approximately 1.5-fold dose shift for an equivalent acute skin response (Yoshida et al., 2025). However, these effects are clearly dependent on irradiation volume, beam arrangement, and delivery parameters and therefore cannot be directly extrapolated to all clinical irradiation scenarios.

#### 3.1.5 Cardiovascular, bone/cartilage, and other systems

Compared with the organ systems discussed above, evidence for FLASH radiotherapy in the cardiovascular system, bone/cartilage, and other tissues remains relatively limited. The available studies are also modality-specific: most tissue-remodeling and head-and-neck data summarized below were obtained with proton beams, whereas the negative lymphocytopenia study used 20-MeV electrons. Therefore, these findings should be interpreted as preliminary tissue-level observations rather than definitive evidence of broad normal-tissue protection. Available data suggest that the potential protective effects of FLASH in these systems are most evident in injury processes characterized by persistent inflammation, fibrosis, and tissue remodeling.

In the cardiovascular system, current evidence primarily suggests that FLASH may attenuate late remodeling after radiation-induced cardiac injury. In a model of focal cardiac proton irradiation, the same dose of 40 Gy was delivered using either FLASH or conventional dose rate proton irradiation. At 2–3 weeks after irradiation, the conventional dose rate group showed greater upregulation of TGF-β1 and α-SMA, whereas expression levels in the FLASH group remained closer to those in nonirradiated controls. These findings suggest that FLASH may reduce profibrotic responses and thereby contribute to longer-term preservation of cardiac function (Kim et al., 2024). However, this protective effect does not appear to extend to all cardiovascular- or immune-related endpoints. In a model of cardiac irradiation–associated lymphocytopenia, FLASH conferred no detectable benefit. Whether delivered as 2 Gy × 5 fractions or as a single 10 Gy fraction, ultra-high dose rate irradiation failed to mitigate peripheral depletion of CD3^+^, CD4^+^, CD8^+^, or CD19^+^ lymphocytes, and recovery was delayed at selected time points (Venkatesulu et al., 2019). An independent Mobetron electron-beam study (>100 Gy/s) likewise found that short-term gonadal toxicity was comparable after ultra-high and conventional dose rate irradiation. Thus, although FLASH may attenuate profibrotic and remodeling-associated responses in cardiac tissue, these effects cannot be directly extrapolated to hematologic or immunologic endpoints.

In bone, cartilage, and adjacent mesenchymal tissues, preliminary evidence suggests that FLASH may reduce muscle atrophy and aberrant bone remodeling after irradiation. In a hindlimb proton irradiation model, 30 Gy FLASH was associated with less severe gastrocnemius atrophy and bone resorption at 27 days after irradiation compared with conventional dose rate irradiation. FLASH also reduced activation of injury-associated pathways related to osteoclast differentiation, endochondral bone morphogenesis, and chondrocyte development. These findings suggest that FLASH may exert tissue-protective effects by attenuating radiation-induced disruption of bone and cartilage remodeling and by limiting degeneration of adjacent soft tissues (Velalopoulou et al., 2021).

Additional evidence comes from head and neck irradiation models. In mice receiving proton irradiation to the head and neck region, FLASH improved survival and reduced injury to the salivary glands and oral mucosa compared with conventional dose rate irradiation, whether delivered as a single dose of 14–18 Gy or as three fractions of 8 Gy. FLASH was also associated with greater preservation of AQP5 expression in the salivary glands, as well as reduced tongue ulceration and late fibrosis (Chowdhury et al., 2024). However, FLASH has not demonstrated clear protective benefits in models of splenic lymphocytopenia or acute gonadal toxicity (Venkatesulu et al., 2019).

Overall, although evidence for FLASH in cardiovascular, bone/cartilage, head and neck, and other organ systems remains less extensive than that for the brain, lung, and gastrointestinal tract, the available findings suggest a recurring pattern: FLASH is more likely to show normal-tissue sparing for endpoints related to fibrosis, chronic inflammation, and tissue remodeling. In contrast, its benefits for preventing acute immune-cell depletion appear variable or absent (Kim et al., 2024).

Across organ systems, the most consistently reported protective effects include reduced acute tissue injury and inflammatory amplification, preservation of regenerative cell populations, decreased persistent DNA damage and cellular senescence, and attenuation of late fibrosis or pathological remodeling. The predominant manifestation differs among tissues. Functional preservation is most evident in the brain, protection of regenerative niches is particularly relevant in the lung and intestine, and reduced superficial tissue injury and contracture are commonly observed in the skin and soft tissues.

Collectively, these findings suggest a shared cross-organ principle: FLASH irradiation may alter the trajectory of the local post-irradiation microenvironment rather than uniformly suppress a single molecular pathway. Specifically, the tissue response may shift from persistent inflammation and maladaptive remodeling toward earlier resolution and improved recovery. However, these effects remain dependent on organ type, radiation modality, linear energy transfer (LET), dose-rate profile, irradiated volume, and follow-up duration. Moreover, they are not consistently observed across all endpoints, particularly acute lymphocyte depletion and injury following large-volume irradiation.

### 3.2 Preservation of tumor control

The tissue microenvironmental changes described in the animal studies above collectively suggest that FLASH radiotherapy can attenuate inflammatory amplification, preserve regenerative capacity, and delay chronic remodeling in selected normal organs. However, these effects cannot be directly extrapolated to tumor tissue. For FLASH to have meaningful translational value, normal-tissue protection must be achieved without compromising antitumor efficacy. In other words, an expanded therapeutic window can only be established if favorable changes in the normal-tissue microenvironment occur concurrently with preserved tumor control.

This issue is particularly important from a microenvironmental perspective. Reduced inflammation, preservation of regenerative capacity, and improved repair responses in normal tissues do not necessarily imply that the tumor microenvironment will respond in the same direction. Tumors may exhibit distinct responses because of intrinsic hypoxia, metabolic stress, immunosuppressive signaling, altered vascular architecture, and heterogeneous radiosensitivity. Therefore, after establishing that FLASH can induce differential microenvironmental effects in normal tissues, it is necessary to determine whether this radiotherapeutic context can maintain local tumor control comparable to that achieved with conventional dose rate radiotherapy in preclinical tumor models. In selected settings, it is also important to assess whether FLASH induces tumor microenvironmental changes that may be therapeutically favorable. Encouragingly, available evidence indicates that tumor control with FLASH has been preserved in several preclinical tumor models, providing a necessary foundation for the transition of FLASH from a normal-tissue-sparing phenomenon to a clinically translatable therapeutic strategy.

#### 3.2.1 Lung cancer and thoracic tumor models

In lung cancer and thoracic tumor models, current preclinical studies generally suggest that FLASH irradiation provides tumor control comparable to that of conventional dose rate radiotherapy. In some orthotopic lung cancer models, FLASH may even induce microenvironmental changes associated with enhanced antitumor activity. Favaudon et al. demonstrated in a syngeneic orthotopic thoracic tumor model that FLASH and conventional dose rate irradiation were similarly effective in inhibiting tumor growth, establishing early evidence that FLASH can preserve tumor control in thoracic tumor models (Favaudon et al., 2014). In an orthotopic Lewis lung carcinoma model that more closely recapitulates the lung cancer microenvironment, Shukla et al. showed that 60 Gy/s proton FLASH delivered using a clinical pencil-beam scanning proton system preserved antitumor efficacy. Moreover, FLASH was associated with reduced tumor proliferative activity and enhanced DNA damage responses, as reflected by fewer Ki-67-positive tumor cells and increased γH2AX-positive cells. These changes were accompanied by increased CD8^+^ T-cell infiltration and reduced immunosuppressive cellular components. Thus, in this orthotopic non–small cell lung cancer model, proton FLASH may enhance antitumor responses by remodeling the immune microenvironment (Shukla et al., 2023). Nevertheless, this finding is currently based on a limited number of thoracic and orthotopic lung cancer studies and requires further validation using standardized irradiation parameters, rigorous local control endpoints, and long-term survival outcomes (Favaudon et al., 2014).

#### 3.2.2 Breast cancer models

Breast cancer models have been used primarily to evaluate whether FLASH preserves local tumor control under rigorous experimental conditions. Sørensen et al. applied proton pencil-beam scanning FLASH in a C3H mouse breast cancer model using Tumor Control Dose 50% (TCD50) as the primary endpoint. The TCD50 values for FLASH and conventional dose rate irradiation were 51.3 Gy and 49.1 Gy, respectively, indicating nearly equivalent local tumor control. Importantly, this study used a curative endpoint rather than relying solely on short-term tumor growth delay, thereby providing stronger evidence that FLASH did not compromise local tumor control (Sørensen et al., 2022).

Melemenidis et al. subsequently compared electron FLASH irradiation at 93–200 Gy/s with conventional dose rate irradiation at 0.08 Gy/s in a Py117 syngeneic orthotopic breast cancer model using single-fraction doses of 20, 25, or 30 Gy. Tumor control was similar between the two dose-rate conditions across dose levels. When irradiation was focused on the abdominal wall and follow-up was extended, the FLASH group achieved a 75% tumor-free survival rate at 48 days. In contrast, larger tumors mainly exhibited growth delay rather than complete eradication (Melemenidis et al., 2025). These findings suggest that, in this nonmetastatic orthotopic breast cancer model, tumor volume and prescribed dose are major determinants of cure probability, whereas dose rate itself does not appear to compromise local tumor control.

Taken together, lung and breast cancer models provide important preclinical support for the concept that FLASH may broaden the therapeutic window while preserving local tumor control comparable to that of conventional dose rate radiotherapy (McGarrigle et al., 2024).

#### 3.2.3 Glioma and glioblastoma models

In brain tumor models, current evidence suggests that the antitumor efficacy of FLASH radiotherapy is broadly comparable to that of conventional dose rate radiotherapy in primary brain tumor settings. In orthotopic glioblastoma models, both FLASH and conventional dose rate irradiation, delivered either as a single fraction of 10–14 Gy or as fractionated whole-brain irradiation of 2 × 7 Gy, significantly reduced tumor bioluminescence and prolonged survival. However, no significant difference in tumor control was observed between the two dose-rate conditions (Montay-Gruel et al., 2021).

n an intracerebral glioblastoma model using immunocompetent rats, single-dose FLASH irradiation at 20–30 Gy produced a dose-response relationship similar to that observed with conventional dose rate irradiation. Doses of 20–25 Gy significantly prolonged survival, whereas 30 Gy further reduced tumor size but did not confer additional survival benefit, suggesting that acute radiation toxicity may become dose limiting at this level (Liljedahl et al., 2023). Overall, FLASH appears to provide tumor control comparable to conventional dose rate irradiation in currently available brain tumor models. However, direct evidence regarding FLASH-mediated tumor control in brain metastasis models remains limited (Shi et al., 2024).

#### 3.2.4 Pancreatic cancer and abdominal tumor models

In pancreatic cancer models, the antitumor efficacy of FLASH appears broadly comparable to that of conventional dose rate irradiation, although definitive evidence of superiority is lacking. In the initial dosimetric validation study of a proton FLASH system, Diffenderfer et al. used MH641905 pancreatic flank tumors derived from a Kras-P53-Pdx1-Cre (KPC) spontaneous pancreatic cancer model to evaluate tumor growth after 12 Gy and 18 Gy proton irradiation. Both FLASH and conventional dose rate proton irradiation significantly inhibited tumor growth and delayed tumor regrowth, with no significant difference between the two modalities. These findings indicate that FLASH preserved at least equivalent tumor control in this pancreatic cancer model (Liljedahl et al., 2023).

Additional evidence comes from a preclinical study of a scattered proton FLASH platform conducted by the University of Washington group. Although this study primarily evaluated normal-tissue injury after whole-pelvis irradiation, the concurrently established B16F10 flank tumor model showed that 18 Gy FLASH achieved flank tumor control comparable to that of conventional dose rate proton irradiation. Although these results are not specific to gastrointestinal tumors, they provide additional methodological support for the conclusion that, in abdominal and pelvic irradiation contexts, ultra-high dose rate delivery does not necessarily reduce local antitumor efficacy (Cao et al., 2024).

Accordingly, existing evidence suggests that FLASH-mediated tumor control in pancreatic and abdominal tumor models is comparable to that achieved with conventional dose rate irradiation. However, this conclusion is based mainly on a limited number of flank tumor models, including pancreatic cancer models. Direct studies of FLASH tumor control in orthotopic pancreatic cancer, colorectal cancer, pelvic tumors, and peritoneal metastasis models remain scarce. Thus, the evidence base in this area remains substantially less developed than that for lung, thoracic, and breast tumor models.

#### 3.2.5 Squamous cell carcinoma model

In squamous cell carcinoma models, current evidence on tumor control with FLASH irradiation is concentrated mainly in head and neck squamous cell carcinoma and oral squamous cell carcinoma. In most radiosensitive models, FLASH and conventional dose rate irradiation appear to achieve comparable tumor suppression. However, in radiation-resistant models, FLASH may have additional therapeutic potential.

Chowdhury et al. used an orthotopic head and neck squamous cell carcinoma mouse model with Mouse Oral Cancer 2 (MOC2) orthotopic tongue tumors to compare single-fraction proton FLASH at 128 Gy/s, delivered at 14–18 Gy or as 8 Gy × 3 fractions, with conventional dose rate proton therapy at 0.95 Gy/s. Both approaches achieved substantial tumor volume reduction. However, the FLASH group showed improved overall survival, which was largely attributable to reduced normal-tissue toxicity rather than clearly superior intrinsic tumor control (Chowdhury et al., 2024).

Recent studies suggest that, in radiation-resistant head and neck squamous cell carcinoma, FLASH may extend beyond simple equivalence. A 2025 study of ultra-high dose rate radiotherapy using high-energy X-rays reported that, in radiation-resistant head and neck squamous cell carcinoma cell lines and animal models, UHDR irradiation had effects comparable to conventional dose rate irradiation in radiosensitive cells but more strongly suppressed proliferation and invasion in radiation-resistant cells. It also enhanced DNA damage and apoptosis. *In vivo*, UHDR irradiation partially reversed radioresistance and was associated with increased CD8^+^ T-cell infiltration and a higher M1/M2 macrophage ratio (Li et al., 2025). However, this finding should currently be regarded as an important model-specific observation rather than a generalizable conclusion. Radiation resistance in head and neck squamous cell carcinoma is multifactorial, involving enhanced DNA repair, hypoxia, tumor stemness, cell-cycle alterations, and immunosuppression, and substantial heterogeneity exists across experimental models (Hutchinson et al., 2020).

#### 3.2.6 Summary of tumor control evidence

Across existing preclinical tumor models, FLASH generally shows no consistent evidence of compromised tumor control in the systems studied to date. This provides a necessary foundation for the concept of therapeutic window expansion, given the differential microenvironmental effects observed in normal tissues. However, this conclusion should be interpreted cautiously.

First, much of the current evidence is based on surrogate endpoints, including tumor growth delay, tumor volume reduction, or decreased bioluminescence. Studies using rigorous local control endpoints, such as TCD50 or long-term tumor-free survival, remain limited. Strictly defined local tumor control should be based on curative endpoints rather than short-term measures of tumor response. Although tumor growth delay, volume reduction, and reduced bioluminescence can reflect short-term antitumor activity, these endpoints are influenced by treatment duration, tumor volume, tumor biology, follow-up length, and endpoint definitions. They therefore cannot be directly equated with tumor cure or uncompromised local control.

Second, the available data are highly heterogeneous with respect to tumor type, beam platform, dose-rate structure, radiation modality, irradiation geometry, and follow-up duration. This heterogeneity is insufficient to support the conclusion that FLASH universally preserves tumor control across all tumor types. Third, whether FLASH can consistently maintain antitumor efficacy in hypoxic tumors, radiation-resistant tumors, or immunosuppressive tumor microenvironments remains unresolved (Loo et al., 2024; Limoli and Vozenin, 2023).

Therefore, the current consensus is that preclinical tumor studies have preliminarily demonstrated the potential of FLASH to preserve tumor control in selected systems. However, this potential requires further validation using more rigorous curative endpoints, more complex orthotopic and immunocompetent models, and standardized parameter frameworks. The translational promise of FLASH depends on two simultaneous conditions: favorable remodeling of the normal-tissue microenvironment and preservation of tumor control. In selected contexts, FLASH may also induce tumor microenvironmental responses distinct from normal-tissue protection that could further optimize antitumor efficacy. Future research should therefore move beyond verifying equivalent tumor control and instead identify the specific tumor microenvironmental conditions under which FLASH is most likely to preserve or enhance antitumor effects.

To improve the comparability, reproducibility, and auditability of preclinical studies, future animal experiments should include rigorously matched FLASH and conventional dose-rate irradiation groups. Radiation modality, beam energy, total dose, fractionation schedule, irradiated volume, and irradiation geometry should be kept consistent between groups. Studies should report the radiation modality, beam energy, linear energy transfer (LET) or LET distribution when applicable, mean dose rate, dose per pulse, pulse duration, pulse repetition frequency, number of pulses, total beam-on time, and methods used for dosimetric verification. For platforms with spatially and temporally nonuniform dose delivery, such as proton pencil-beam scanning, local dose-rate distributions and relevant scanning parameters should also be reported (Böhlen et al., 2024; Zou et al., 2023). Tumor growth delay, tumor-volume reduction, and changes in bioluminescence may be included as secondary endpoints but should not be used alone to establish equivalent tumor control. Whenever feasible, curative endpoints, including the tumor control dose required to achieve local control in 50% of tumors (TCD50), long-term local control, recurrence, and disease-free survival, should be prioritized. The follow-up period should be sufficiently long to capture delayed recurrence (Konradsson et al., 2022). Studies should also include orthotopic and immunocompetent tumor models, particularly models with well-characterized hypoxic or radioresistant phenotypes. Prespecified subgroup analyses should account for tumor type, baseline tumor volume, oxygenation status, radiation modality, LET, and dose-rate profile. A conclusion that FLASH irradiation does not compromise tumor control should be supported by formal noninferiority analyses based on curative endpoints, such as long-term local control or TCD50.

## 4 Early clinical translation

Based on the animal studies discussed above, FLASH radiotherapy induces tissue-level microenvironmental changes across multiple organ systems. These changes are associated with attenuated inflammatory amplification, preservation of regenerative capacity, reduced chronic fibrosis, and improved long-term functional maintenance. At the same time, selected preclinical tumor models suggest that antitumor efficacy is not substantially compromised under FLASH conditions. Therefore, the purpose of clinical translation is to identify therapeutic scenarios in which this potential therapeutic window is most likely to be realized—that is, settings in which favorable normal-tissue microenvironmental remodeling can be achieved while tumor control is preserved.

Current clinical studies indicate that FLASH translation remains primarily in the stage of feasibility and early safety validation. The focus of investigation is gradually shifting from whether ultra-high dose rate delivery can be technically achieved to determining in which disease settings, anatomical sites, and dose conditions a clinically meaningful expansion of the therapeutic window can be reproducibly observed. Accordingly, early clinical studies have prioritized scenarios such as superficial skin lesions and painful bone metastases in the extremities. These settings offer relatively controllable treatment geometry, greater feasibility of *in vivo* dosimetric verification, more readily observable normal-tissue responses, and tumor control assessments that are less likely to be confounded by complex conformal planning factors. These choices reflect the underlying logic of FLASH clinical development: initial human studies should first test whether the preclinical normal-tissue-sparing phenotype can be translated into clinically observable benefits in settings most favorable for detecting differential local tissue responses.

The design of current FLASH clinical studies is largely consistent with this translational rationale. As summarized in Table 3, the most advanced clinical investigations have focused on superficial tumors and palliative treatment of bone metastases. Other disease sites and organ systems remain largely in stages of trial preparation, platform development, or protocol optimization, and mature clinical outcome data remain limited (Mascia et al., 2023; Kinj et al., 2024; Bourhis et al., 2019).

**TABLE 3:** Early clinical translation of FLASH-RT.

Disease system	Indication	Key study/trial	Radiation modality	Dose/fractionation	Design/phase	Main endpoints	Key takeaway	Registry	References
Skin/superficial tumors	Cutaneous T-cell lymphoma (refractory skin lesion)	First-in-human e-FLASH	Electron (e-FLASH)	15 Gy × 1	Case report (first-in-human)	Feasibility and safety; acute skin reaction; tumor response	Demonstrated clinical feasibility of UHDR electron delivery with acceptable normal-skin response (short follow-up)	—	Bourhis et al. (2019)
Skin/superficial tumors	Cutaneous lymphoma (two lesions in one patient)	Intra-patient comparison: UHDR vs. conventional dose rate	Electron (UHDR vs. conventional)	15 Gy × 1 (both lesions)	Intra-patient controlled case study	Acute/late toxicity and local control (2-year follow-up)	No major differences between UHDR and conventional dose rate, highlighting context-dependence and need for standardization	—	Gaide et al. (2022)
Skin/superficial tumors	Localized cutaneous BCC/SCC (radical intent)	LANCE randomized phase II selection trial protocol	Electron (e-FLASH vs. conventional)	T1: 22 Gy × 1 T2: 30 Gy/5 fx (protocol)	Randomized, open-label phase II (protocol)	Primary: ≥G3 toxicity (6 weeks); Secondary: local control (12 months)	First randomized radical-intent framework to test clinical benefit of FLASH vs. conventional RT in skin cancer	NCT05724875	Kinj et al. (2024)
Skin/superficial tumors	Melanoma skin metastases	Flash-Skin I: prospective phase I implementation and RTQA report	Electron (hybrid e-FLASH + conventional)	2 × 9 Gy UHDR +1 × 9 Gy conventional (hybrid)	Prospective phase I (implementation/RTQA; n = 7)	Feasibility, reproducibility, and QA; safety	Shows reproducible clinical LINAC-based e-FLASH workflow and RTQA in a small cohort (step toward scalable evidence generation)	NCT06549439	Dal Bello et al. (2026)
Skin/superficial tumors	Melanoma skin metastases (dose exploration)	IMPulse: dose-escalation phase I study	Electron (e-FLASH)	Dose escalation	Phase I, single-center dose escalation (ongoing)	Safety, feasibility, dose finding	Represents ongoing dose-finding efforts to define a clinically usable parameter window in melanoma skin metastases	NCT04986696	Bourhis, (2023)
Musculoskeletal (palliation)	Symptomatic extremity bone metastases (pain palliation)	FAST-01: first-in-human proton FLASH trial	Proton (p-FLASH, transmission beam)	8 Gy × 1	Prospective non-randomized feasibility/safety (n = 10)	Workflow feasibility; toxicity; pain response	Demonstrated clinical workflow feasibility with mostly mild toxicities and pain-relief signals comparable to standard palliation (small sample)	NCT04592887	Mascia et al. (2023)
Musculoskeletal (palliation)	Thoracic bone metastases (near heart/lung; pain palliation)	FAST-02 protocol	Proton (p-FLASH)	Single-fraction palliation	Prospective protocol study	Toxicity; pain response; workflow and QA metrics	Extends p-FLASH palliation to more complex anatomy to test safety/robustness near critical organs	NCT05524064	Daugherty et al. (2024)
Other systems (emerging)	Deep-seated poor-prognosis cancers (platform development)	FRATHEA FLASH-VHEE platform	Very high-energy electrons (VHEE-FLASH) and PBS FLASH	—	Platform build-out/clinical trial preparation	—	No mature patient outcome data yet; illustrates the translational roadmap for deep targets and multi-angle access	—	—
Skin/superficial tumors	Superficial skin tumors	Phase I safety protocol	Electron (e-FLASH)	Protocol-defined superficial irradiation	Phase I protocol	Toxicity and preliminary efficacy	Expands prospective e-FLASH evaluation beyond isolated case reports	ChiCTR2400080935	Yang et al. (2025b)
Emerging X-FLASH platform	Conformal treatment of deeper targets	CHEXs/MAX-FLASH systems	High-energy X-rays	Compact source; multiangle delivery under development	Preclinical platform development	Dose rate; conformality; image guidance	Addresses accessibility and multiangle delivery; mature patient outcome data are not yet available	—	Liu et al. (2025a), Lin et al. (2025)

*BCC: basal cell carcinoma; SCC: squamous cell carcinoma; RTQA: radiotherapy quality assurance; LINAC: linear accelerator; QA: quality assurance; VHEE: Very High-Energy Electrons; PBS: Pencil-Beam Scanning.

### 4.1 Skin system

FLASH radiotherapy was first translated clinically in cutaneous lesions, where superficial tumor geometry, shallow electron penetration, relatively simple field configuration, and feasible dosimetric verification provided favorable conditions for the safe implementation of ultra-high dose rate irradiation. In this setting, clinical development has progressed stepwise from first-in-human feasibility assessment to within-patient comparative observations and, more recently, to randomized trial designs with standardized treatment parameters.

The first human application of FLASH radiotherapy, reported in 2019, provided an important proof of concept for clinical translation in cutaneous disease. A single fraction of 15 Gy was delivered over an ultrashort treatment time using a low-energy electron beam. Dose delivery and *in vitro* dosimetric validation were performed under patient-specific treatment conditions. The treatment was feasible and safe, with favorable normal-skin tolerance and tumor response (Bourhis et al., 2019). Although this study did not provide mechanistic evidence, it represented a critical initial step in clinical translation by demonstrating that FLASH irradiation could be delivered to a patient under quality-controlled conditions and could generate an evaluable clinical response. It therefore established the feasibility basis for subsequent systematic clinical studies.

Within-patient comparative evidence in cutaneous disease has also provided important information for clinical translation. In one patient with cutaneous lymphoma, two independent lesions were treated on the same day with the same prescribed dose of 15 Gy in a single fraction, using either conventional dose rate irradiation at approximately 0.08 Gy/s or ultra-high dose rate irradiation at approximately 166 Gy/s. After 2 years of follow-up, no major differences were observed in acute toxicity, late toxicity, or tumor control between the two lesions (Gaide et al., 2022). This observation suggests that even in superficial cutaneous lesions, which are among the most technically favorable indications for FLASH, additional normal-tissue sparing may be context dependent and influenced by physical beam parameters, treatment geometry, and underlying tissue biology. Accordingly, clinical translation of FLASH is moving beyond the question of whether the effect can be observed toward defining the specific parameter and disease contexts in which clinically meaningful benefit can be reproducibly demonstrated.

The cutaneous field has now advanced toward randomized evaluation of clinical benefit. LANCE (NCT05724875) is a phase II trial comparing FLASH radiotherapy with conventional radiotherapy in a radical-intent setting for patients with localized cutaneous basal cell carcinoma or squamous cell carcinoma (T1–T2, N0M0). The protocol specifies low-energy electron FLASH delivery at approximately 220–270 Gy/s, with a tumor-size–adapted prescription strategy of 22 Gy in a single fraction for T1 disease and 30 Gy in five fractions for T2 disease. It also defines an evaluation framework focused on clinically relevant endpoints, including toxicity and local control (Gaide et al., 2022). This randomized design moves cutaneous FLASH beyond anecdotal or small-sample clinical experience and into an evidence-based comparative framework. It enables formal assessment of whether FLASH can reduce toxicity while maintaining, or potentially improving, local tumor control under predefined eligibility criteria, reproducible parameter ranges, and standardized endpoint evaluation.

In parallel, the randomized Flash-Skin I trial (NCT06549439) was designed to evaluate trial feasibility and reproducibility. A prospective phase I study including seven patients with melanoma skin metastases used a hybrid regimen consisting of two fractions of 9 Gy delivered at ultra-high dose rate followed by one fraction of 9 Gy delivered at conventional dose rate. This study reported implementation details and quality assurance procedures on a clinical linear accelerator platform (Dal Bello et al., 2026). These findings suggest that electron FLASH can be implemented in patients and that adaptation of conventional linear accelerators for electron FLASH may facilitate broader multicenter clinical testing, thereby accelerating evidence generation and indication expansion (von der Grün et al., 2026). Collectively, these clinical efforts indicate that cutaneous FLASH is feasible, auditable, and potentially reproducible within standard clinical quality assurance systems.

Early clinical translation in dermatologic indications has therefore established feasibility and provided the first framework for comparative clinical testing. However, existing studies remain at an early stage and are limited by small sample sizes, heterogeneous clinical scenarios, and immature long-term outcome data. Consequently, current evidence is insufficient to establish definitive clinical efficacy, and further randomized studies with standardized dosimetry, longer follow-up, and clinically meaningful toxicity and tumor-control endpoints are required.

### 4.2 Musculoskeletal system

Another clinical application progressing in parallel with cutaneous FLASH is the treatment of bone metastases within the musculoskeletal system. In this setting, the initial objective has been to evaluate the feasibility and safety of proton FLASH in a palliative context with relatively low clinical risk, using a single-fraction analgesic prescription of 8 Gy. The nonrandomized FAST-01 trial enrolled patients with painful bone metastases in the extremities and delivered 8 Gy in a single fraction using single-beam transmission proton therapy at an ultra-high dose rate of at least 40 Gy/s. The study demonstrated clinical feasibility, showed predominantly mild treatment-related adverse events, and reported an analgesic response signal comparable to that expected with conventional dose rate palliative radiotherapy in a limited patient cohort (Mascia et al., 2023). This study represents the first transition of FLASH from a laboratory-defined dosimetric concept to a clinical workflow incorporating treatment prescription, outcome assessment, and follow-up procedures similar to routine palliative radiotherapy practice.

FAST-02 builds on FAST-01 by extending the clinical evaluation of proton FLASH to thoracic bone metastases, which are anatomically closer to critical organs such as the lung and heart. The protocol prioritizes toxicity and pain-response outcomes while incorporating workflow and quality assurance metrics to evaluate the stability, reproducibility, and safety of FLASH delivery in more complex anatomical regions (Mascia et al., 2023; Ghita-Pettigrew et al., 2026).

More broadly, the major clinical advance of FLASH to date is its transition from a preclinical concept to human application. Procedural feasibility and early safety have been preliminarily demonstrated, primarily for electron FLASH in superficial or cutaneous lesions and proton FLASH for palliation of painful bone metastases. FAST-01 demonstrated the feasibility and preliminary safety of proton FLASH in 10 patients with extremity bone metastases, with pain-relief outcomes in this small cohort appearing broadly comparable to those historically reported for conventional dose rate palliative radiotherapy. Meanwhile, studies such as the LANCE randomized phase II trial and FAST-02 indicate that FLASH clinical development is moving from case-based feasibility assessment toward prospective comparative trial designs (von der Grün et al., 2026; Mascia et al., 2023; Kinj et al., 2024; Bourhis et al., 2019; Daugherty et al., 2024).

Animal and preclinical studies similarly suggest that the FLASH effect is not consistently reproducible across all experimental conditions. In a mouse model of localized abdominal irradiation, proton FLASH delivered at approximately 120 Gy/s did not reduce intestinal injury or lymphopenia; some endpoints even suggested increased toxicity (Zh et al., 2023). In a mouse model of gonadal irradiation, the acute toxicity induced by ultra-high-dose-rate (UHDR) electron irradiation was generally comparable to that observed after conventional-dose-rate irradiation, with no clear evidence of a protective effect (Cuitiño et al., 2023). Studies in cats with nasal squamous cell carcinoma and in minipigs have further shown that, although FLASH irradiation may mitigate some early skin reactions, severe late necrosis can still occur after large-volume irradiation (Rohrer Bley et al., 2022).

Collectively, these findings indicate that exceeding a specific mean dose-rate threshold alone is insufficient to ensure a reproducible FLASH effect. Several physical and biological factors may influence the response. First, dose per pulse, instantaneous dose rate, pulse interval, and total beam-on time collectively affect oxygen depletion, radical recombination, and subsequent oxidative damage over short timescales. When the dose is delivered in multiple pulses or the overall irradiation time is prolonged, the tissue-sparing effect may be attenuated (Karsch et al., 2022). Second, different radiation modalities and delivery techniques produce distinct spatial and temporal dose distributions. This issue is particularly relevant to proton pencil-beam scanning, in which local dose rate, irradiation depth, and linear energy transfer (LET) vary across the irradiated volume. Consequently, the nominal mean dose rate may not accurately reflect the conditions experienced by individual tissue regions. Recent studies have also reported inconsistent findings for acute gastrointestinal toxicity following proton and electron UHDR irradiation (Liu K. et al., 2025).

The irradiated volume, fractionation schedule, and biological model are also important determinants of the response. Large-volume irradiation may affect a broader range of regenerative niches and vascular compartments, whereas fractionated irradiation may alter reoxygenation, DNA repair, and immune-cell recruitment. Organ-specific factors, including cell turnover rate, baseline oxygenation, and the timing of outcome assessment, may further influence whether a protective effect is detected. Thus, negative findings do not necessarily invalidate the FLASH effect. Rather, they indicate that its occurrence depends on irradiation parameters, organ type, and the biological model.

## 5 Microenvironmental changes induced by FLASH-RT

The microenvironmental effects induced by FLASH radiotherapy are not uniform. Instead, they appear to reflect a remodeling process that is dependent on irradiation parameters, tissue context, and postirradiation time (Velalopoulou et al., 2021; Petusseau et al., 2024). Under conditions of ultra-high dose rate and very short delivery time, initial radiochemical processes, oxygen-dependent reactions, and radical kinetics may be altered. These early events provide a plausible shared upstream framework, but their magnitude is expected to differ across electron, X-ray, proton, and carbon-ion beams because their microdosimetric energy-deposition patterns and track structures are not equivalent. They may subsequently influence multiple components of the tissue microenvironment, including redox status, inflammatory amplification, barrier integrity, regenerative repair, fibrotic progression, mitochondrial homeostasis, and immune ecosystem remodeling.

On this basis, this section summarizes the principal microenvironmental modules that may be affected by FLASH, including oxidative stress and the reactive oxygen species environment, inflammatory cell death and tissue-barrier integrity, delayed injury and fibrosis, the DNA damage response, mitochondrial and metabolic homeostasis, immune regulation, and the cGAS–STING/type I interferon axis. These modules should not be considered in isolation. Rather, they collectively constitute an interconnected framework through which FLASH may alter the normal trajectory of tissue injury, repair, tumor response, and therapeutic outcome. Importantly, the evidence is organized at two levels: potentially shared processes, such as altered oxygen-dependent chemistry and oxidative burden, and modality-specific downstream observations that still require cross-beam validation (Table 4).

**TABLE 4:** Evidence boundaries of FLASH-induced microenvironmental changes across radiation modalities.

Evidence level	Microenvironmental alteration	Representative evidence and beam type	Interpretation and extrapolation boundary
Potentially shared	Altered oxygen-dependent reactions and radical-recombination kinetics	Direct comparison of X-rays, protons, and carbon ions showed that oxygen consumption depends on dose, dose rate, and LET; complete oxygen depletion was not observed at clinically relevant doses (Jansen et al., 2021)	A plausible upstream physicochemical framework across modalities. However, track structure and LET differ among beams; complete oxygen depletion should not be presented as the sole mechanism
	Reduced oxidative stress and secondary radical-mediated injury	Proton-FLASH irradiation of IMR90 cells reduced ROS elevation and preserved mitochondrial function. Low-LET electron lung models also indicated less early and persistent injury (Guo et al., 2022; Fouillade et al., 2020)	Reduced oxidative burden may be a recurring downstream outcome. A single antioxidant pathway should not be assumed for all beams because tissue oxygenation, LET, and pulse structure may modify the response
	Reduced persistent DNA damage and cellular senescence	Low-LET electron lung models showed fewer persistent DNA-damage foci and less replicative senescence. Electron whole-abdominal irradiation also reduced injury to crypt base columnar cells (Fouillade et al., 2020; Levy et al., 2020)	A recurring cellular-injury endpoint in normal-tissue sparing. Cross-modality validation remains incomplete, and identical DNA-damage spectra or repair kinetics should not be assumed
	Attenuated inflammatory amplification	Electron lung, X-ray intestinal, and proton cardiac models reported attenuation of inflammation-related changes, although the implicated cell populations and signaling pathways differed (Fouillade et al., 2020; Shi et al., 2022; Kim et al., 2024)	Attenuation of tissue-level inflammation may represent a shared pattern. Specific immune pathways should still be described according to beam type, organ, and time window
	Reduced fibrotic remodeling and preservation of regenerative niches	Different forms of sparing were reported in lung, skin, soft tissue, heart, and intestine, including lung-progenitor preservation after electron irradiation, enhanced crypt regeneration after electron irradiation, and attenuated cardiac fibrotic pathways after proton irradiation (Fouillade et al., 2020; Levy et al., 2020; Kim et al., 2024)	A recurring tissue-level sparing pattern across organs. Regenerative cell populations and fibrotic endpoints remain organ dependent; cross-organ and cross-beam extrapolation requires qualification
Beam or model specific	Attenuation of the cytosolic dsDNA-cGAS-STING-CD8+ T-cell chemotaxis-GSDME pyroptosis axis	Directly demonstrated in a whole-abdominal FLASH X-ray mouse model combined with PD-L1 blockade (Shi et al., 2022)	A relatively well-defined mechanism in an X-ray intestinal model. It should not be presented as a universal immune mechanism of FLASH; applicability to electrons, protons, and carbon ions remains to be tested
	Drp1-related regulation of mitochondrial dynamics and preservation of mitochondrial function	Observed in proton-FLASH-irradiated normal human lung fibroblasts (IMR90): 15 Gy, approximately 100 Gy/s, and LET approximately 10 keV/μm (Guo et al., 2022)	Current evidence is mainly derived from a proton-beam cellular model. Direct extrapolation to other radiation modalities or in vivo organs is not justified
	Preservation of lung progenitors with reduced persistent DNA damage and replicative senescence	Directly observed in a low-LET 4.5-MeV electron lung-irradiation model (Fouillade et al., 2020)	An important tissue-sparing mechanism in an electron-beam lung model. It cannot yet be assumed to apply to proton, X-ray, or high-LET carbon-ion lung irradiation
​	Preservation of crypt stem cells, enhanced regeneration, and improved barrier integrity	Consistently supported in low-LET high-energy electron whole-abdominal models (Levy et al., 2020; Ruan et al., 2021)	A reproducible histological endpoint in electron-beam intestinal models. It should not be generalized to all proton or carbon-ion abdominal models; protection also depends on dose rate and pulse structure
	Attenuation of TGF-β1-, α-SMA-, and collagen-associated cardiac remodeling	Observed in a focal cardiac proton-FLASH model, together with reduced inflammation and preserved cardiac function (Kim et al., 2024)	Evidence of attenuated fibrotic remodeling in a proton cardiac model. These signaling changes should not be generalized to all organs or radiation modalities
	LET- and dose-rate-dependent sparing with high-LET carbon ions	Carbon-ion abdominal irradiation showed stronger sparing at high LET when dose rate exceeded 100 Gy/s; entrance-region hindlimb irradiation also reduced acute skin reactions (Cao et al., 2025; Yoshida et al., 2025)	The sparing window of high-LET carbon ions has an independent parameter dependence. Conclusions from low-LET electrons or X-rays should not be transferred directly; entrance, Bragg-peak, and SOBP regions require separate validation

### 5.1 Oxygen microenvironment

**FIGURE 3:**
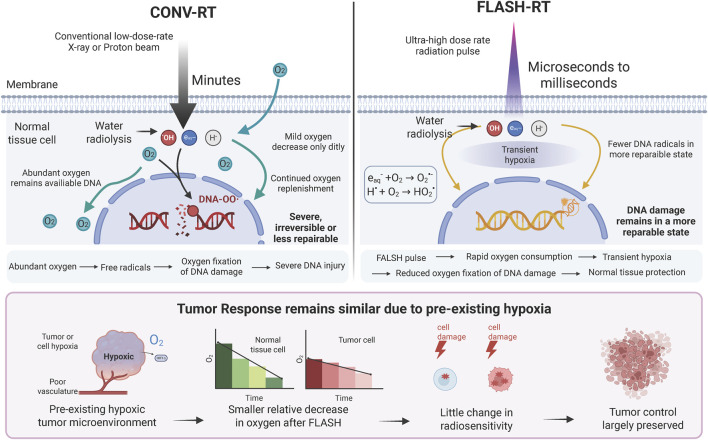
Schematic overview of oxygen-related mechanisms proposed for the FLASH effect. A schematic comparison of conventional radiotherapy and FLASH radiotherapy illustrating how ultrafast dose delivery may alter oxygen-dependent radiation chemistry, reduce oxygen fixation of DNA damage, promote normal tissue sparing, and yet preserve tumor control because of pre-existing tumor hypoxia.

The oxygen microenvironment is an important modulator of the FLASH effect, but it should not yet be regarded as a definitively established central mechanism. Because ultra-high dose rate irradiation is delivered within milliseconds or even shorter time scales, it may theoretically alter the extent to which oxygen participates in early radiation-chemical reactions, thereby influencing DNA damage fixation, peroxide formation, and subsequent oxidative injury (Figure 3) (Boscolo et al., 2021). However, oxygen consumption and oxygen sensitivity are not expected to be identical across beam types. They depend on dose, pulse structure, track structure, and LET. Current evidence therefore supports a more cautious interpretation: although oxygen tension may modulate the magnitude and direction of the FLASH effect, transient hypoxia caused solely by radiolytic oxygen depletion (ROD) is insufficient to explain all instances of FLASH-mediated normal-tissue protection across different tissues and irradiation conditions.

Montay-Gruel et al. reported in a mouse whole-brain electron-irradiation irradiation model that carbogen inhalation, which increases tissue oxygen tension, attenuated the neuroprotective effect of FLASH. This finding suggests that oxygenation may contribute to biological differences between FLASH and conventional dose rate irradiation (Boscolo et al., 2021; Montay-Gruel et al., 2019). However, these observations were derived from a specific low-LET electron-beam brain irradiation models, and independent reproducible validation across other organs, species, and irradiation conditions remains limited. Therefore, these data are better interpreted as evidence that oxygenation contributes to the FLASH effect, rather than as direct confirmation that ROD is the established underlying mechanism. This distinction is important because carbogen inhalation not only changes tissue pO2 but may also alter perfusion, metabolism, and inflammatory status. Thus, mechanistic conclusions drawn from this pharmacologic and physiologic intervention remain inherently limited.

From a quantitative perspective, the ROD hypothesis also faces important constraints. *In vivo* electron paramagnetic resonance measurements showed that, after a 20 Gy FLASH dose, oxygen partial pressure decreased by approximately 2.3 mmHg in normal tissue and by approximately 1.0 mmHg in tumor tissue. In addition, direct measurements in water phantoms irradiated with 225 kV X-rays, 224 MeV protons, and carbon ions showed that oxygen consumption varied with dose rate and LET, but complete oxygen depletion was not observed at clinically relevant doses (Jansen et al., 2021). These findings suggest that, at the tissue level, FLASH-induced oxygen consumption is generally insufficient to shift well-oxygenated tissue into a hypoxic range with substantial radiobiological relevance (Cao et al., 2021). Other experimental and modeling studies have similarly suggested that, at clinically relevant doses of approximately 10 Gy, oxygen depletion is typically limited to only a few mmHg. Such a decrease is unlikely, by itself, to explain the robust normal-tissue protection observed with FLASH, particularly in tissues that are normoxic or only mildly hypoxic (Cao et al., 2021). Therefore, discussions of the oxygen hypothesis should consider both the magnitude of ROD and the pO2 range required to produce a meaningful change in the oxygen enhancement ratio (OER). Normal-tissue protection cannot be inferred solely from the occurrence of oxygen consumption.

Tumor control represents an additional critical issue. If ROD were sufficient to substantially reduce oxygen-mediated damage fixation, one direct implication would be that FLASH could further reduce tumor-cell radiosensitivity in already hypoxic tumor regions. This possibility would conflict with the translational objective of sparing normal tissues without compromising tumor control. Therefore, the oxygen hypothesis cannot be used solely to explain normal-tissue protection; it must also account for tumor responses under different oxygenation states, particularly whether antitumor efficacy may be reduced in hypoxic tumors.

On the basis of current evidence, a more plausible interpretation is that oxygenation is an important regulatory factor in the FLASH effect, whereas ROD itself is only one component of a broader network of radiochemical and biological processes. FLASH may simultaneously influence reactive oxygen species/reactive nitrogen species kinetics, hydrogen peroxide production, lipid oxidation, mitochondrial stress, immune and inflammatory amplification, and programmed cell death (Scarmelotto et al., 2024). However, most of these mechanisms remain supported primarily by hypotheses or indirect evidence and should not be presented as definitive conclusions. Future studies will require real-time or near-real-time measurement approaches, including *in vivo* and *ex vivo* pO2 monitoring, electron paramagnetic resonance, fluorescence- or mass spectrometry–based detection of oxidative damage, pulse radiolysis, reactive oxygen species kinetic analysis, multiscale computational modeling, and dosimetric validation aligned with dose rate, pulse structure, and LET. Such approaches are necessary to establish causal relationships among oxygen dynamics, radical reactions, and downstream biological effects. Mechanistic studies of FLASH should therefore be grounded in direct experimental measurements and computational modeling, rather than relying solely on phenomenologic outcomes to infer reactive oxygen species dynamics, oxygen kinetics, or radical recombination mechanisms (Ali and El Naqa, 2025).

### 5.2 Free radicals and the reactive oxygen species microenvironment

**FIGURE 4:**
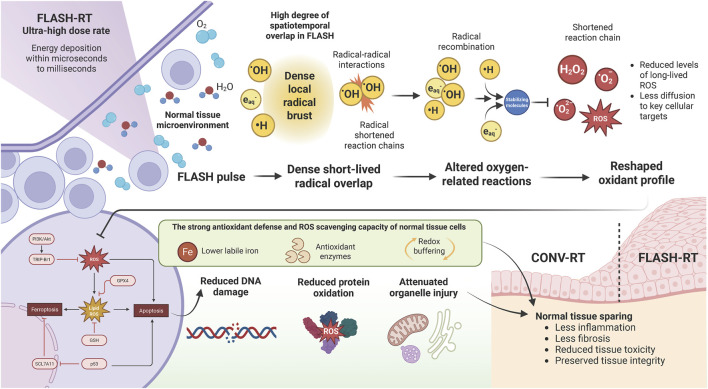
Schematic model of FLASH-induced radical overlap, oxidant profile reshaping, and normal tissue protection. FLASH irradiation may reduce the effective oxidative damage burden by promoting short-lived radical overlap and self-termination of reaction chains, thereby limiting long-lived ROS accumulation and downstream molecular and tissue injury in normal tissues.

The free-radical and reactive oxygen species (ROS) microenvironment provides a critical link between early radiation chemistry and downstream tissue responses. The existing free-radical/peroxyl-radical complexation hypothesis proposes that FLASH irradiation generates high transient radical concentrations within an extremely short time window, thereby promoting complexation or recombination between organic radicals and peroxyl radicals. This process may shorten lipid peroxidation chain reactions and reduce persistent oxidative injury. On the basis of reaction-kinetics modeling, Labarbe et al. proposed that Peroxyl radical (ROO·) complexation may represent a key chemical mechanism underlying FLASH-mediated normal-tissue protection (Figure 4) (Labarbe et al., 2020). This concept is potentially relevant across modalities, but the extent of radical overlap cannot be assumed to be identical for beams with different LET and track structures.

However, this model is still based primarily on homogeneous chemical reactions and parameter fitting and cannot substitute for direct measurements or simulations of realistic track structures. Ionizing radiation initially produces ionization events and water-radiolysis products along particle tracks. Whether these radicals undergo recombination depends on their spatial separation, lifetime, and diffusion range at nanometer-to-micrometer scales. Therefore, LET, particle type, track structure, and intertrack spacing are fundamental physical parameters that must be incorporated into discussions of ROS kinetics. Both high dose rate and high LET can increase local radical density, but through different mechanisms: high dose rate primarily increases interactions between separate tracks, whereas high LET enhances radical reactions within individual tracks (Jay-Gerin, , 2025). This distinction is particularly important across radiation modalities. Even at the same average dose rate, electron FLASH, X-ray FLASH, and proton FLASH differ in LET distribution, secondary-electron spectra, track density, and probability of intertrack overlap (Audouin et al., 2023; Audouin et al., 2025). Recent carbon-ion studies further indicate that tissue sparing can vary with LET and dose rate, reinforcing the need for modality-specific interpretation (Cao et al., 2025). Therefore, ROS-related findings obtained with one FLASH beam modality cannot be directly extrapolated to all forms of FLASH irradiation.

Current simulation results regarding intertrack radical interactions are also inconsistent. Some Monte Carlo studies suggest that, within certain FLASH parameter ranges, intertrack interactions may be insufficient to explain the observed FLASH effects. However, these models remain constrained by assumptions regarding radical diffusion, chemical reaction networks, and spatial scale definitions (Baikalov et al., 2023). Thus, radical recombination and ROS profile modulation remain attractive mechanistic hypotheses, but their validity depends on LET, particle type, pulse structure, dose per pulse, total irradiation time, and the local tissue oxygenation state. Future studies should integrate Monte Carlo track-structure simulations, pulse radiolysis, electron paramagnetic resonance, fluorescence-based radical probes, lipid oxidation omics, and rigorous dosimetric validation. Such approaches are needed to determine whether radical recombination constitutes a common mechanism of FLASH-mediated normal-tissue protection and to define the actual free-radical and ROS microenvironment generated by FLASH under different irradiation conditions.

### 5.3 Inflammatory microenvironment

The potential benefits of FLASH radiotherapy for normal tissues extend beyond reduced initial injury and may include attenuation of injury-induced inflammatory amplification. Radiation-induced endothelial injury can trigger inflammatory cell death, disrupt tissue barriers, and promote immune-cell infiltration, thereby establishing a positive feedback loop that amplifies local damage into organ-level toxicity. Within this framework, the potential value of FLASH may lie in shifting irradiated tissues toward a more repair-permissive state, thereby alleviating acute toxicity and reducing subsequent chronic remodeling, including fibrosis (Ruan et al., 2021).

Recent evidence supporting a role for inflammatory cell death in FLASH-mediated tissue protection was reported by Shi et al., in 2022. In a mouse model of anti-PD-L1–combined radiotherapy, ultra-high dose rate FLASH X-rays markedly reduced intestinal toxicity. The study identified a mechanistic cascade involving cytoplasmic double-stranded DNA accumulation, cGAS–STING activation, immune-effector enhancement, gasdermin-mediated pyroptosis, and subsequent crypt injury and barrier disruption. Under FLASH conditions, this cascade was attenuated in intestinal tissue, resulting in reduced crypt pyroptosis and preservation of barrier integrity. These findings provide direct mechanistic evidence that FLASH may improve the tolerability of combined radiotherapy and immune checkpoint blockade (Shi et al., 2022).

Radiation-induced inflammatory cell-death pathways, including the NLRP3 inflammasome-associated pyroptotic axis, have also been implicated in radiation injury, providing an independent mechanistic basis for further investigation (Wu et al., 2018). In future translational studies, measurable readouts such as alveolar survival, tissue permeability, IL-1β and IL-18 levels, and GSDMD/GSDME cleavage could be systematically assessed to establish a closed-loop evidence framework linking irradiation temporal structure, inflammatory cell death, and clinical toxicity (Kim et al., 2024).

Importantly, reduced cGAS–STING signaling should not be regarded as a universally beneficial effect or a consistent feature of FLASH irradiation. In a FLASH X-ray model combined with PD-L1 blockade, attenuated cGAS–STING activation protected normal intestinal crypts from inflammation-associated injury, while cGAS-dependent antitumor immunity was preserved in tumor tissues (Shi et al., 2022). These findings underscore the spatially distinct and context-dependent roles of the same signaling pathway in normal tissues and tumors.

### 5.4 TGF-β–mediated fibrosis and late toxicity

The major clinical burden of radiation injury often manifests as late structural damage, including excessive extracellular matrix (ECM) deposition, increased tissue stiffness, and progressive loss of organ function. In multiple organs, the TGF-β/SMAD pathway is considered a central mediator linking initial radiation-induced injury signaling to subsequent fibrotic remodeling. Radiation-induced activation of TGF-β promotes fibroblast differentiation into myofibroblasts, stimulates collagen and fibronectin deposition, and is further amplified by persistent oxidative stress and chronic inflammation. Therefore, TGF-β signaling represents a critical and potentially targetable component of radiation-induced fibrosis (Martin et al., 2000).

In the context of FLASH radiotherapy, delayed functional benefits have been observed in radiosensitive organs such as the lung and heart (Fouillade et al., 2020). In murine thoracic irradiation models, ultra-high dose rate FLASH irradiation markedly reduced advanced pulmonary fibrosis and related late sequelae compared with conventional dose rate irradiation, suggesting that ultrashort dose delivery may alter the early trajectory toward chronic tissue remodeling (Favaudon et al., 2014; Luo et al., 2026). Cardiac models provide additional evidence. Studies of radiation-induced cardiac injury after proton FLASH irradiation indicate that FLASH can attenuate inflammatory and profibrotic markers, particularly TGF-β–related signaling, while better preserving cardiac function and reducing long-term collagen deposition and fibrosis compared with conventional dose rate irradiation. Similar assessments using photon FLASH have also examined cardiac functional decline, remodeling, and myocardial fibrosis at late time points, although the strength and consistency of these findings require further clarification (Kim et al., 2024). These observations suggest that, by reducing the early inflammatory burden, FLASH may limit amplification of downstream fibrotic pathways and thereby mitigate late clinical toxicity.

A plausible interpretation is that the effects of TGF-β signaling after FLASH irradiation vary across disease stages and tissue compartments (Flechsig et al., 2012). During the acute injury phase, transient activation of TGF-β/EMT-related signaling in alveolar epithelial cells may facilitate epithelial adaptation and tissue repair. By contrast, sustained activation of the canonical TGF-β/SMAD pathway in fibroblasts and myofibroblasts during the later remodeling phase promotes extracellular matrix deposition and fibrosis. These findings are not necessarily contradictory, because the biological consequences of TGF-β signaling depend on the duration of pathway activation, the responding cell type, and the tissue compartment involved. Nevertheless, repair-associated EMT should not be regarded as uniformly beneficial. Persistent TGF-β/EMT signaling in the tumor microenvironment may increase tumor cell plasticity, invasion, treatment resistance, and metastatic potential (Martin et al., 2000). Future studies should characterize the temporal dynamics of TGF-β signaling in paired normal-tissue and tumor models and determine whether early repair-associated responses subside before pathological remodeling begins.

### 5.5 DNA damage response and repair kinetics

**FIGURE 5:**
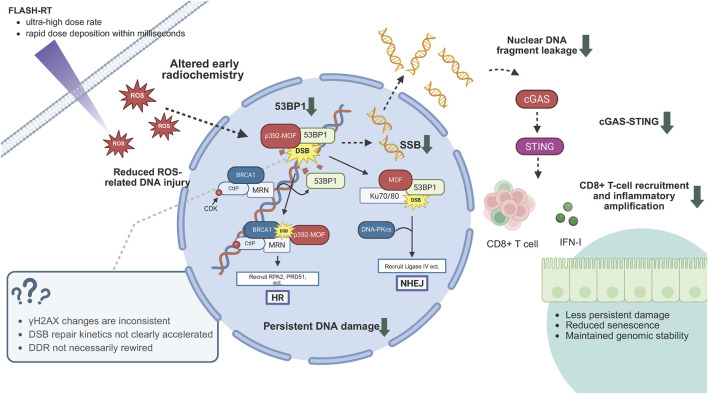
Schematic model of FLASH-associated DNA integrity preservation and attenuation of the cytosolic dsDNA–cGAS–STING inflammatory axis. This schematic illustrates a DDR-centered but upstream-driven model of FLASH normal tissue sparing. Ultra-high dose rate FLASH irradiation alters early radiochemistry and reduces ROS-related DNA injury, leading to fewer 53BP1-associated lesions, reduced single-strand breaks, less persistent DNA damage, and decreased leakage of nuclear DNA fragments into the cytoplasm. As a consequence, activation of the cGAS–STING pathway, type I interferon signaling, and CD8^+^ T-cell recruitment/inflammatory amplification are attenuated, thereby contributing to reduced senescence, maintained genomic stability, and normal tissue protection. The figure also highlights current uncertainties, including inconsistent γH2AX findings, the lack of clear evidence for accelerated classical DSB repair kinetics, and the possibility that DDR itself is not fundamentally rewired by FLASH.

The DNA damage response (DDR) is central to radiation biology. It governs the recognition and repair of radiation-induced DNA lesions, including DNA double-strand breaks (DSBs), and influences cell survival, genomic stability, and long-term tissue function after irradiation. Current evidence suggests that the principal differences associated with FLASH are more likely to arise from earlier radiochemical events than from a consistent reprogramming of canonical DDR pathways. Specifically, FLASH may indirectly alter DNA damage profiles by modulating the reactive oxygen species burden and oxidative injury cascade, although whether DDR signaling itself is consistently reshaped remains uncertain (Friedl et al., 2022). As illustrated in Figure 5, the current working model places the main FLASH-associated divergence upstream of DDR: altered early radiochemistry may reduce ROS-associated DNA injury, decrease persistent DNA damage, and limit downstream inflammatory signaling triggered by cytosolic DNA leakage.

Several studies have suggested that ultra-high dose rate irradiation does not substantially alter the kinetics of classical DSB repair. Experiments comparing ultra-high dose rate proton irradiation with conventional dose rate X-ray irradiation, using 53BP1 foci as a surrogate marker, showed comparable resolution of 53BP1 foci over time. These findings indicate that DSB repair is not intrinsically accelerated or delayed under ultra-high dose rate conditions (Hanton et al., 2019). Nevertheless, other studies suggest that the damage profile, rather than the repair rate, may differ under FLASH conditions, with trends toward reduced DNA damage burden or decreased clustered DNA damage (Perstin et al., 2022; Sforza et al., 2024). These findings are consistent with the emerging view that differences in early radiochemistry and tissue microenvironmental responses may reduce the burden of damage requiring repair, whereas DDR functions primarily as a downstream response to upstream injury and as a mediator linking DNA damage to cell death and inflammation. Figure 5 further summarizes this concept by highlighting reduced 53BP1-associated damage, decreased leakage of nuclear DNA fragments into the cytosol, and weaker activation of the cGAS–STING inflammatory axis. It also emphasizes unresolved issues, including inconsistent γH2AX findings and the lack of clear evidence that classical DSB repair kinetics are systematically accelerated by FLASH.

### 5.6 Mitochondrial homeostasis and metabolic reprogramming

Radiation injury affects not only the nucleus and plasma membrane but also mitochondria, which play central roles in cellular energy metabolism, redox buffering, cell-death regulation, and inflammatory signaling. Therefore, if FLASH alters upstream reactive oxygen species kinetics and oxidative burden, these changes may be further amplified or buffered by mitochondrial responses, thereby influencing apoptosis, inflammatory signaling, and tissue repair. Recent mechanistic studies suggest that preservation of mitochondrial function may contribute to FLASH-mediated normal-tissue protection and may be closely linked to mitochondrial dynamics (Guo et al., 2022).

Guo et al. compared FLASH proton irradiation at approximately 100 Gy/s with conventional dose rate proton irradiation in normal human lung fibroblasts (IMR90 cells) and reported that mitochondrial activity and cellular proliferation were better preserved under FLASH conditions. The study identified mitochondrial dynamic homeostasis, regulated in part by phosphorylation of dynamin-related protein 1 (Drp1), as a potential contributor to this effect. These findings suggest that maintenance of balanced mitochondrial fission and quality-control processes may be an important component of normal-cell resistance to radiation-induced stress (Guo et al., 2022).

Caggiano et al. further showed in a pancreas-associated model that FLASH preserved mitochondrial function and reduced mitochondrial stress in normal pancreatic tissues compared with conventional dose rate irradiation. However, this mitochondrial protection was not observed in pancreatic ductal adenocarcinoma (PDAC), suggesting that mitochondrial integrity may contribute selectively to normal-tissue protection rather than uniformly protecting tumor tissue (Caggiano et al., 2025). Additional studies of mitochondria-mediated apoptosis and inflammatory signaling indicate that alterations in mitochondrial membrane permeability, cytochrome c release, and downstream signaling pathways, including interferon-β–related responses, may exhibit distinct regulatory patterns under ultra-high dose rate conditions (Lv et al., 2025).

Collectively, these findings support the hypothesis that mitochondrial homeostasis may represent an important downstream interface between early FLASH-related radiochemical changes and subsequent biological outcomes. However, whether mitochondrial preservation is a primary driver of FLASH-mediated normal-tissue sparing or a secondary consequence of reduced upstream oxidative injury remains unresolved. Future studies should integrate mitochondrial functional assays, metabolic profiling, redox measurements, and paired tumor–normal tissue analyses to clarify the role of mitochondrial dynamics and metabolic reprogramming in FLASH radiobiology.

Mitochondrial responses should also be interpreted in a time- and cell-specific context. Early preservation of mitochondrial integrity may reduce oxidative damage and apoptosis in normal cells. By contrast, sustained alterations in mitochondrial dynamics or mtDNA-associated inflammatory signaling may have different biological consequences in tumor cells (Lv et al., 2025). In addition, evidence regarding Drp1 has been derived primarily from proton-irradiated fibroblast models and should not yet be extrapolated to other radiation modalities (Guo et al., 2022).

### 5.7 Immune regulation and innate immune pathways

The effects of FLASH radiotherapy on the immune response may be partly related to the substantial reduction in irradiation time. Shorter delivery times may reduce the dose absorbed by circulating blood as it repeatedly traverses the irradiation field, thereby lowering the risk of radiation-induced lymphopenia (RIL) (Chabi et al., 2021). Delivering radiation within an ultrashort time window can reduce both the probability that circulating immune cells are exposed to the beam and the cumulative dose received by these cells, thereby producing a potential immune-cell-sparing effect (Jin et al., 2020). Time-dependent blood-flow and dose-modeling studies have systematically estimated the fraction of circulating lymphocytes exposed to irradiation and the corresponding depletion rate under FLASH conditions. These models suggest that pencil-beam scanning FLASH may markedly reduce irradiation and depletion of circulating lymphocytes compared with conventionally fractionated intensity-modulated proton therapy under specific fractionation and dose-rate conditions (Galts and Hammi, 2024). Therefore, FLASH delivery over very short time intervals may reduce unnecessary immune-cell depletion and help preserve systemic immune competence and treatment tolerability.

Merging evidence also suggests that FLASH may reshape the immune microenvironment in tumors and tumor-draining lymph nodes (TDLNs). Compared with conventional dose rate irradiation, FLASH has been associated with greater retention of activated T cells in tumors and TDLNs, accompanied by increased intratumoral infiltration of CD8^+^ T cells and higher expression of immune checkpoint markers, including PD-1 and PD-L1 ^44^. Increased T-cell retention and infiltration suggest that FLASH may enhance postirradiation antitumor immune responses in selected contexts. At the same time, upregulation of checkpoint molecules indicates that FLASH-induced immune activation may provide a biological rationale for combination strategies involving immune checkpoint inhibitors, particularly agents targeting the PD-1/PD-L1 axis (Chen et al., 2024; Eggold et al., 2022; Lyu et al., 2025).

## 6 Current microenvironment-based combination strategies

Combination strategies based on FLASH-induced microenvironmental remodeling remain at an early exploratory stage. The rationale is not simply to add another therapeutic modality, but to exploit the potential advantages of FLASH—including normal-tissue sparing, redox modulation, immune microenvironment remodeling, and alterations in the residual cellular ecosystem associated with recurrence—to enhance tumor control or reduce recurrence risk while preserving the normal-tissue protective window.

The most concentrated evidence currently involves nanomedicine and local delivery platforms. These systems can spatially confine immunostimulants, photothermal agents, metabolic inhibitors, or ferroptosis-inducing components to the tumor site, thereby aligning with the central objective of FLASH: expansion of the therapeutic window. Current efforts primarily seek to use nanotechnology platforms to amplify tumor-selective FLASH responses while maintaining normal-tissue protection. Representative strategies include induction of ferroptosis through Pd–C single-atom nanozymes, carbon monoxide release, and glutathione depletion in combination with immunotherapy; use of AIEgen-modified TaOx nanospheres or biomimetic membrane-encapsulated systems to enhance clearance of cancer stem cells after FLASH, thereby reducing recurrence and metastatic potential; delivery of CB-839 and the mild photothermal agent TPE-BBT through thermosensitive hydrogels to combine metabolic inhibition, photothermal therapy, and interference with DNA repair; and use of AuNP-IMQ hydrogels to achieve FLASH-triggered release of immunomodulators for melanoma treatment (Lyu et al., 2023a; Suo et al., 2024; Dong et al., 2023; Shen et al., 2024; Lyu et al., 2023b).

From a microenvironmental perspective, these strategies are not intended merely to intensify the delivered radiation effect. Rather, they aim to target tumor-protective mechanisms that may persist after FLASH irradiation. For example, ferroptosis-based strategies primarily target tumor redox homeostasis and lipid peroxidation vulnerability; cancer stem cell–elimination strategies target the residual cell ecosystem and recurrence-associated microenvironment after radiotherapy; photothermal and metabolic inhibition strategies aim to weaken tumor repair capacity and metabolic adaptation; and imiquimod-loaded hydrogels primarily modulate the local immune microenvironment. These studies suggest that nanomedicine may serve as an important bridge between the physical advantages of FLASH and targeted intervention in the tumor microenvironment.

Evidence is also emerging for combinations of FLASH with cell-based therapies, such as chimeric antigen receptor T-cell therapy. FLASH radiotherapy has been reported to reverse immunosuppression by reprogramming lipid metabolism in tumor-associated macrophages, thereby promoting chimeric antigen receptor T-cell infiltration and activation and enhancing the sensitivity of medulloblastoma to GD2-directed chimeric antigen receptor T-cell therapy (Saenz et al., 2025; Lira et al., 2025; Portier et al., 2024). This line of evidence suggests that FLASH may not only preserve immune-cell viability but also reshape the tumor immune microenvironment through innate immune and metabolic pathways, thereby improving the efficacy of immunotherapy (Kim et al., 2021).

In 2022, Eggold et al. investigated the therapeutic potential of abdominopelvic FLASH irradiation combined with PD-1 blockade. In a mouse model of ovarian cancer, FLASH radiotherapy promoted intestinal regeneration while maintaining tumor control. The combination reduced tumor burden, accompanied by decreased intratumoral regulatory T cells and increased cytotoxic CD8^+^ T cells. Compared with conventional dose rate radiotherapy, FLASH increased intratumoral T-cell infiltration and maintained the capacity to enhance the efficacy of anti-PD-1 therapy (Eggold et al., 2022).

However, the limitations of current combination strategies are substantial. First, the number of studies remains limited, and available evidence is concentrated in a small number of preclinical models, with insufficient validation across tumor types, irradiation platforms, and long-term safety endpoints. Second, many nanoscale systems have complex compositions, making it difficult to isolate the specific contribution of FLASH-induced microenvironmental remodeling from the independent effects of the delivery platform or therapeutic payload. Third, although existing studies emphasize tumor suppression and reduced recurrence, they often insufficiently evaluate whether combination strategies compromise normal-tissue protection, whether the FLASH parameter window remains valid, and what long-term immunologic and toxicologic consequences may arise.

Therefore, current FLASH-based combination strategies remain in the preclinical proof-of-concept stage. More systematic studies integrating mechanistic validation, dosimetric standardization, and safety assessment are required before clinical translation. Identifying measurable biomarkers, druggable intervention points, and optimal sequencing strategies based on FLASH-induced microenvironmental changes will help move FLASH research beyond primarily phenomenologic observations toward a more mechanism-driven and translationally verifiable stage. Potential microenvironment-based intervention targets and combination strategies for future research are summarized in Table 5.

**TABLE 5:** Potential intervention targets and strategies based on the FLASH microenvironment effect.

Combination module	Representative strategies	Rationale for combination with FLASH-RT	Current evidence base	FLASH-specific translational readiness	Required evidence	References
DDR inhibition	PARP, ATR, and DNA-PK inhibitors	Blocks tumor DNA damage repair and increases replication stress and double-strand break persistence; a strong radiosensitization rationale already exists in conventional RT.	High for non-FLASH RT combinations; early clinical data exist for PARPi/ATRi/DNA-PKi with RT, but FLASH-specific evidence remains limited	Highest priority for FLASH-specific de-risking; not ready for direct extrapolation from conventional RT to FLASH clinical trials	Head-to-head CONV, FLASH, drug + CONV, and drug + FLASH testing; tumor control plus acute/late normal-tissue toxicity; schedule, fractionation, PK/PD; γH2AX, 53BP1, RAD51, replication stress, and tissue repair assays	Javed et al. (2024), Samuels et al. (2024), Fulton et al. (2018), Luo et al. (2025)
Immune checkpoint blockade	Anti-PD-1/PD-L1 and anti-CTLA-4	FLASH may reduce gastrointestinal or marrow toxicity while preserving or enhancing radiation-induced antitumor immunity	Moderate: extensive conventional RT + ICI experience and FLASH + PD-1 evidence in an abdominopelvic ovarian cancer model, but FLASH models remain limited	Early candidate in selected indications and anatomical sites	Immunocompetent and multiple tumor models; fractionation; local control, abscopal effects, CD8^+^ T cells, Treg, myeloid cells, cytokines, and normal-tissue immune toxicity	Eggold et al. (2022), Colangelo et al. (2024)
Conventional chemotherapy radiosensitizers	Cisplatin, temozolomide, 5-FUetc.	Leverages established chemoradiotherapy principles to increase tumor killing	Moderate for conventional RT; weak for FLASH-specific combinations. Drug toxicity may erode normal-tissue sparing	Should be evaluated drug by drug rather than as a unified module	Demonstrate that the drug does not abolish FLASH sparing; assess marrow, GI, lung, and brain dose-limiting toxicities; compare drug-radiation intervals	Javed et al. (2024), Samuels et al. (2024), Fulton et al. (2018)
Oxygen/redox modulation	Carbogen, hypoxia modulation, antioxidants, ROS/RNS modulators	Attempts to modulate oxygen fixation, ROD, ROS kinetics, lipid peroxidation, and radical chemistry	Low and contested: ROD, radical recombination, and ROS kinetics are largely model-based or indirectly inferred, with insufficient real-time *in vivo* measurements	Mechanistic research module; not ready as a clinical combination strategy	Require *in vivo*/*ex vivo* pO2 monitoring, EPR, pulse radiolysis, ROS/RNS kinetics, lipid peroxidation assays, and multiscale modeling; test whether hypoxic tumor control is compromised	Ali and El Naqa (2025), Boscolo et al. (2021)
Inflammation/cGAS-STING/pyroptosis	STING modulators, inflammasome or pyroptosis-pathway modulators	FLASH may reduce cytoplasmic dsDNA accumulation, cGAS-STING activation, T-cell chemotaxis, and GSDME-mediated pyroptosis, thereby limiting secondary inflammatory amplification	Low-to-moderate: supported by intestinal FLASH radioimmunotherapy models, but mainly in specific tissues and acute toxicity settings	Mechanistically promising but requires de-risking	Separate normal-tissue protection from potential weakening of antitumor immunity; assess tumor IFN-I/STING signaling, CD8^+^ activation, barrier injury, and inflammatory cell death	Shi et al. (2022), Colangelo et al. (2024)
Mitochondrial/metabolic modulation	Mitochondrial ROS modulators, metabolic reprogramming, NRF2/antioxidant pathways	May influence post-FLASH oxidative stress, cell death, and tissue repair	Low: mostly hypothetical or indirect, with limited FLASH-specific causal validation	Exploratory stage	Measure mitochondrial ROS, membrane potential, metabolic flux, ATP, and NADH/NAD+; use genetic/pharmacologic perturbation to establish causality for sparing and tumor control	Ali and El Naqa (2025), Wang et al. (2025)
Nanomedicine/radiosensitizers	High-Z nanoparticles, nanozymes, targeted delivery, immune nanomedicines	Can enhance local tumor dose deposition, modulate ROS/GSH, induce ferroptosis, or deliver combination agents	Low-to-moderate: conventional RT nanoradiosensitization evidence is broader; FLASH-specific work is mainly proof-of-concept, with some nanozyme-based FLASH radioimmunotherapy studies	Not ready for direct clinical translation unless safety, pharmacokinetics, and dosimetry are well characterized	Validate UHDR dose enhancement, ROS yield, biodistribution, clearance, and long-term safety; prove that FLASH normal-tissue sparing is preserved after combination	Lyu et al. (2023b), Wang et al. (2025), Bilynsky et al. (2022)
Tumor microenvironment targeting	Hypoxia, vasculature, TGF-β, myeloid cells, CAF modulation	Uses potential differential FLASH effects on vascular, inflammatory, immune, and stromal microenvironments	Low: conceptually plausible but fragmented; most data come from conventional RT or FLASH-alone observations	Exploratory stage	Integrate single-cell, spatial transcriptomic, imaging, and functional assays; compare whether FLASH and CONV drive the same or opposite microenvironmental changes in tumor and normal tissues; confirm tumor killing is not reduced	Favaudon et al. (2014), Ali and El Naqa (2025), Montay-Gruel et al. (2017), Wang et al. (2025)

*RNS: reactive nitrogen species; ICI: immune checkpoint inhibitor; CAF: Cancer-Associated Fibroblast.

## 7 Conclusion

Research on the FLASH microenvironment has shifted from early explanations based on a single mechanism toward a comprehensive understanding of the continuous chain linking the physical-chemical and radiation environments with the tissue microenvironment. The central message of this review is that FLASH-RT should be understood as a context-dependent process of microenvironmental remodeling across a physical-chemical-biological continuum. Beam characteristics and radiochemical reactions establish the initial conditions, whereas tissue-specific cellular responses determine whether these early events translate into normal-tissue protection, preserved tumor control, or inconsistent outcomes (Ma et al., 2024; Scarmelotto et al., 2024).

Current animal studies indicate that FLASH can mitigate damage in models of the brain, lungs, intestines, skin, and heart; more precisely, it may alter the trajectory of the post-injury tissue microenvironment, including attenuating inflammation, preserving regenerative capacity, and reducing fibrosis and chronic remodeling. Across organs, the most reproducible pattern is not uniform suppression of a single pathway, but a shift from sustained injury amplification toward earlier resolution and more effective tissue recovery. However, this pattern is not universal: its magnitude depends on radiation modality, LET, pulse structure, fractionation, irradiation volume, biological model, and observation time. Negative or inconsistent findings should therefore be interpreted as important boundary conditions rather than as secondary exceptions (Yang T. et al., 2025; Wen et al., 2025).

A second major conclusion is that normal-tissue sparing must always be evaluated together with durable tumor control. Short-term tumor shrinkage or growth delay alone is insufficient to establish therapeutic benefit, particularly in hypoxic, immunosuppressive, or radioresistant tumors. Future studies should prioritize paired tumor-normal tissue models and curative endpoints, such as long-term local control, recurrence-free survival, or TCD50 when feasible.

However, certain shortcomings remain. First, the mechanistic evidence remains scattered, and a unified causal chain has not yet been established between oxygen depletion, ROS, the immune system, mitochondria, and lipid peroxidation. Second, there are significant variations among studies in terms of dose rate, pulse structure, total delivery time, radiation type, and study endpoints, making it difficult to compare results across studies. These limitations define five high-priority directions: (1) mechanistic validation using time-resolved and spatially resolved measurements; (2) standardized and auditable dosimetry, including complete reporting of beam temporal structure and LET-related parameters; (3) systematic evaluation of fractionated FLASH regimens; (4) development of predictive biomarkers that link early microenvironmental changes to late toxicity and tumor control; and (5) stepwise clinical translation through multicenter quality assurance, longer follow-up, and prospective trials.

Finally, many changes in the microenvironment are still inferred indirectly from markers such as inflammatory factors, histological fibrosis, or the proportion of immune cells, and there is a lack of dynamic evidence from real-time measurements of oxygen partial pressure, ROS profiles, metabolic flux, and spatial immunomics. Integrating *in vitro*, *ex vivo*, and *in vivo* models with computational simulation, single-pulse dosimetry, oxygen imaging, and spatial multi-omics will be essential for distinguishing causal mechanisms from correlated observations (Ali and El Naqa, 2025).

Overall, the translational value of FLASH-RT will depend not simply on whether a FLASH effect can be observed, but on whether it can be reproduced under clearly defined conditions, predicted by measurable biomarkers, and implemented without compromising tumor control. A microenvironment-centered framework provides a practical basis for linking physical delivery parameters to clinically meaningful biological outcomes and for guiding the next stage of FLASH research (Vilaplana-Lopera et al., 2025; Romano et al., 2022).
